# Viscoelasticity and accretive phase-change at finite strains

**DOI:** 10.1007/s00033-025-02434-9

**Published:** 2025-01-30

**Authors:** Andrea Chiesa, Ulisse Stefanelli

**Affiliations:** 1 University of Vienna, Faculty of Mathematics and Vienna School of Mathematics, Oskar-Morgenstern-Platz 1, A-1090 Vienna, Austria; 2 University of Vienna, Faculty of Mathematics, Oskar-Morgenstern-Platz 1, A-1090 Vienna, Austria; 3 University of Vienna, Vienna Research Platform on Accelerating Photoreaction Discovery, Währingerstraße 17, 1090 Wien, Austria; 4 Istituto di Matematica Applicata e Tecnologie Informatiche E. Magenes, via Ferrata 1, I-27100 Pavia, Italy

**Keywords:** Accretive growth, Viscoelastic solid, Finite-strain, Viscous evolution, Variational formulation, Viscosity solution, Existence, 74F99, 74G22, 49L25

## Abstract

We investigate the evolution of a two-phase viscoelastic material at finite strains. The phase evolution is assumed to be irreversible: One phase accretes in time in its normal direction, at the expense of the other. Mechanical response depends on the phase. At the same time, growth is influenced by the mechanical state at the boundary of the accreting phase, making the model fully coupled. This setting is inspired by the early stage development of solid tumors, as well as by the swelling of polymer gels. We formulate the evolution problem by coupling the balance of momenta in weak form and the growth dynamics in the viscosity sense. Both a diffused- and a sharp-interface variant of the model are proved to admit solutions and the sharp-interface limit is investigated.

## Introduction

This paper is concerned with the evolution of a viscoelastic compressible solid undergoing phase-change. We assume that the material presents two phases, of which one grows at the expense of the other by *accretion*. In particular, the phase-transition front evolves in a *normal* direction to the accreting phase, with a growth rate depending on the deformation. This behavior is indeed common to different material systems. It may be observed in the early stage development of solid tumors [6, 28, 57], where the neoplastic tissue invades the healthy one. Swelling in polymer gels also follows a similar dynamics, with the swollen phase accreting in the dry one [31, 51] and causing a volume increase. Accretive growth can be observed in some solidification processes [44, 54], as well.

The focus of the modelization is on describing the interplay between mechanical deformation and accretion. On the one hand, the two phases are assumed to have a different mechanical response, having an effect on the viscoelastic evolution of the medium. On the other hand, the time-dependent mechanical deformation is assumed to influence the growth process, as is indeed common in biomaterials [21], polymeric gels [58], and solidification [45]. The mechanical and phase evolutions are thus fully coupled.

**Fig. 1:**
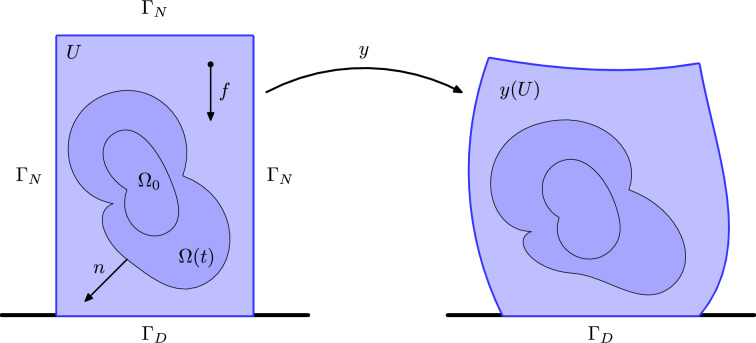
Illustration of the notation in the reference domain (left) and in the deformed one (right)

The state of the system is described by the pair $(y,\theta ): [0,T] \times U \rightarrow \mathbb {R}^d \times [0,\infty )$, where $T>0$ is some final time and $U \subset \mathbb {R}^d\, ( d\ge 2 )$ is the reference configuration of the body. Here, *y* is the deformation of the medium while $\theta$ determines its phase. More precisely, for all $t\in [0,T]$ the *accreting (growing) phase* is identified as the sublevel $\Omega (t):=\{x \in U \ | \ \theta (x)<t\}$, whereas the *receding phase* corresponds to $U {\setminus } \overline{\Omega (t)}$. The value $\theta (x)$ formally corresponds to the time at which the point $x\in U$ is added to the growing phase. As such, $\theta$ is usually referred to as *time-of-attachment* function. An illustration of the notation is given in Fig. 1.

As growth processes and mechanical equilibration typically occur on very different time scales, we neglect inertial effects and assume the evolution to be viscoelastic. This calls for specifying the stored energy density $W(\theta (x){-}t,\nabla y)$ and the viscosity $R(\theta (x){-}t,\nabla y,\nabla \dot{y})$ of the medium, as well as the applied body forces $f(\theta (x){-}t,x)$. All these quantities are assumed to be dependent on the phase via the *sign* of $\theta (x){-}t$, which indeed distinguishes the two phases, in the spirit of the celebrated *level-set method* [41, 46]. In addition, we include a *second-gradient* regularization term in the energy of the form $H(\nabla ^2y)$, which we take to be phase independent, for simplicity. All in all, the viscoelastic evolution system takes the form

1.1 $$\begin{aligned}  -\operatorname {div}\left( \partial _{\nabla y}W (\theta (x){-}t, \nabla y) +\partial _{\nabla \dot{y}}R(\theta (x){-}t,\nabla y,\nabla \dot{y})-\operatorname {div}\textrm{D} H(\nabla ^2y)\right) = f(\theta (x){-}t,x). \end{aligned}$$

This system is solved weakly, complemented by mixed boundary conditions on *y* and a homogeneous natural condition on the *hyperstress* $\textrm{D} H(\nabla ^2 y)$, namely,

1.2 $$\begin{aligned}&y=\textrm{id} \ \ \text {on} \ \ [0,T]\times \Gamma _D, \end{aligned}$$

1.3 $$\begin{aligned}&\textrm{D}H(\nabla ^2y) {:} (\nu \otimes \nu )=0 \ \ \text {on} \ \ [0,T]\times \partial U,\end{aligned}$$

1.4 $$\begin{aligned}&\left( \partial _{\nabla y} W (\theta (x){-}t, \nabla y){+}\partial _{\nabla \dot{y}}R_\varepsilon (\theta (x){-}t,\nabla y, \nabla \dot{y})\right) \nu \nonumber \\&\quad -\textrm{div}_S\,(\textrm{D}H(\nabla ^2 y) \nu ) =0 \ \ \text {on} \ \ [0,T]\times \Gamma _N, \end{aligned}$$

where $\nu$ is the outer unit normal to $\partial U$, $\Gamma _D$ and $\Gamma _N$ are the Dirichlet and Neumann part of the boundary $\partial U$, respectively, and $\textrm{div}_S$ denotes the surface divergence on $\partial U$ [36].

The viscoelastic evolution system is coupled to the phase evolution by requiring that the time-of-attachment function $\theta$ solves the generalized eikonal equation

1.5 $$\begin{aligned} \gamma \big (y(\theta (x) \wedge T ,x),\nabla y(\theta (x) \wedge T ,x)\big )|\nabla (-\theta )(x)|=1 \end{aligned}$$

for all *x* in the complement of a given initial set $\Omega _0\subset \subset U$ where we set $\theta =0$. This corresponds to assuming that $\Omega (t)$ accretes in its normal direction, with *growth rate* $\gamma (\cdot )>0$. More precisely, the evolution of the generic point *x*(*t*) on the boundary $\partial \Omega (t)$ follows the ODE flow

$$\begin{aligned} \frac{\textrm{d}}{\textrm{d} t} x(t) = \gamma \big (y(t,x(t)), \nabla y(t,x(t))\big ) \, \nu (x(t)) \end{aligned}$$

where $\nu (x(t))$ indicates the normal to $\partial \Omega (t)$ at *x*(*t*). Accretive growth is paramount to a wealth of different biological models [50], including plants and trees [15, 17] and the formation of hard tissues like horns or shells [37, 47, 52]. The dependence of the growth rate $\gamma$ on the actual position and strain is intended to model the possible influence of local features such as nutrient concentrations, as well as of the local mechanical state [21]. Note that accretive-growth occurs in a variety of nonbiological systems, as well. These include crystallization [29, 56], sedimentation of rocks [18], glacier formation, accretion of celestial bodies [8], as well as technological applications, from epitaxial deposition [32], to coating, masonry, and 3D printing [20, 30], just to mention a few.

By assuming smoothness and differentiating the equation $\theta (x(t))=t$ in time one obtains $\nabla \theta (x(t)){\cdot }\frac{\textrm{d}}{\textrm{d} t} x(t)=1$. This, together with the above flow rule for *x*(*t*) and $\nu (x(t)) = \nabla \theta (x(t))/|\nabla \theta (x(t))|$, originates the generalized eikonal equation (1.5). As the growth rate $\gamma$ in (1.5) depends on the actual deformation $y(\theta (x) \wedge T,x)$ and strain $\nabla y(\theta (x) \wedge T,x)$ at the growing interface, system (1.1)–(1.5) is fully coupled.

We specify the initial conditions for the system by setting

1.6 $$\begin{aligned}&\theta = 0 \ \ \text {on} \ \Omega _0, \end{aligned}$$

1.7 $$\begin{aligned}&y(0,\cdot ) = y_0 \ \ \text {on} \ U, \end{aligned}$$

where the initial deformation $y_0$ and the initial portion of the growing phase $\Omega _0$ are given. Note that $\Omega _0$ is not required to be connected, in order to possibly model the onset of the accreting phase at different sites. On the other hand, the evolution described by (1.5) does not preserve the topology and disconnected accreting regions may eventually coalesce over time.

The aim of this paper is to present an existence theory to the initial and boundary value problem (1.1)–(1.7). We tackle both a *sharp-interface* and a *diffused-interface* version of the model, by tuning the assumptions on *W* and *R*, see Sects. 2.3–2.4. More precisely, in the diffused-interface model we assume that energy and dissipation densities change smoothly as functions of the phase indicator $\theta (x){-}t$ across a region of width $\varepsilon >0$, namely for $-\varepsilon /2<\theta (x)-t<\varepsilon /2$. On the contrary, in the sharp-interface case material potentials are assumed to be discontinuous across the phase-change surface $\{\theta (x)=t\}$.

In both regimes, we prove that the fully coupled system (1.1)–(1.7) admits a weak/viscosity solution, see Definition 2.1. More precisely, the viscous evolution (1.1)–(1.4) is solved weakly, whereas the growth dynamics equation (1.5) is solved in the viscosity sense, see Theorem 2.1. We moreover prove that solutions fulfill the energy equality, where the energetic contribution of the phase-transition is characterized, see Proposition 2.1. As a by-product, solutions of the diffused-interface model for $\varepsilon >0$ are proved to converge up to subsequences to solutions of the sharp-interface model as the parameter $\varepsilon$ converges to 0, see Corollary 2.1.

Before going on, let us mention that the engineering literature on growth mechanics is vast. Without any claim of completeness, we mention [48, 59] and

[34, 38, 39, 49, 53] as examples of linearized and finite-strain theories, respectively. On the other hand, mathematical existence theories in growth mechanics are scant, and we refer to [3, 12, 19] for some recent results. To the best of our knowledge, no existence result for finite-strain accretive-growth mechanics is currently available. In the linearized case, an existence result for the model [59] has been obtained in [13].

The paper is structured as follows. Section 2 is devoted to the statement of the main existence result, Theorem 2.1. In Sect. 3, we give the proof of the energy identity. The proof of Theorem 2.1 is then split in Sects. 4 and 5, respectively, focusing on the diffused-interface and the sharp-interface setting. In the diffused-interface case, the proof relies on an iterative construction, where the mechanical and the growth problems are solved in alternation. The existence proof for the sharp-interface model is obtained by taking the limit as $\varepsilon \rightarrow 0$ in the diffused-interface model.

## Main results

In this section, we specify assumptions, introduce the weak/viscosity notion of solution, and state the main results for problem (1.1)–(1.7).

### Notation

In what follows, we denote by $$\mathbb {R}^{d\times d}$$ the Euclidean space of $$d{\times } d$$ real matrices, $$d \ge 2 $$, by $$\mathbb {R}^{d\times d}_\textrm{sym}$$ the subspace of symmetric matrices, and by *I* the identity matrix. Given $$A\in \mathbb {R}^{d\times d}$$, we indicate its transpose by $$A^\top $$ and its Frobenius norm by $$ |A|^2:= A{:}A$$, where the contraction product between matrices $$A,\,B\in \mathbb {R}^{d\times d}$$ is defined as $$A {:} B:= A_{ij}B_{ij}$$ (we use the summation convention over repeated indices). Analogously, let $$\mathbb {R}^{d\times d\times d}$$ be the set of real 3-tensors, and define their contraction product as $$A \smash {\vdots } B:= A_{ijk}B_{ijk}$$ for $$A,B\in \mathbb {R}^{d\times d\times d}$$. A 4-tensor $$\mathbb {C}\in \mathbb {R}^{d\times d\times d\times d}$$ is said to be *major symmetric* if $$\mathbb {C}_{ijk\ell } = \mathbb {C}_{k\ell ij}$$ and *minor symmetric* if $$\mathbb {C}_{ijk\ell } = \mathbb {C}_{ij\ell k}=\mathbb {C}_{jik\ell }$$. Given a major and minor symmetric positive definite 4-tensor $$\mathbb {C}\in \mathbb {R}^{d\times d\times d\times d}$$ and the matrix $$A\in \mathbb {R}^{d\times d}$$ we indicate by $$\mathbb {C}{:}A\in \mathbb {R}^{d\times d}$$ and $$ A{:}\mathbb {C}\in \mathbb {R}^{d\times d}$$ the matrices given in components by $$(\mathbb {C}{:}A)_{ij} = \mathbb {C}_{ijk\ell }A_{k\ell }$$ and $$(A{:}\mathbb {C})_{ij} = A_{k\ell }C_{k\ell i j}$$, respectively. We shall use the following matrix sets $$SO(d):=\{ A \in \mathbb {R}^{d\times d} \;|\; \det {A}=1 \ \text {and} \ A A^{\top } =I\}$$ and $$\textrm{GL}_{+}(d):=\{ A \in \mathbb {R}^{d\times d} \;|\; \det A >0 \}$$.

The scalar product of two vectors $a,\, b\in \mathbb {R}^d$ is classically indicated by $a{\cdot }b$. The symbol $B_R\subset \mathbb {R}^{d}$ denotes the open ball of radius $R>0$ and center $0\in \mathbb {R}^{d}$, |*E*| indicates the Lebesgue measure of the Lebesgue-measurable set $E\subset \mathbb {R}^d$, and $\mathbbm {1}_E$ is the corresponding characteristic function, namely, $\mathbbm {1}_E(x)=1$ for $x\in E$ and $\mathbbm {1}_E(x )=0$ otherwise. For $E \subset \mathbb {R}^d$ nonempty and $x\in \mathbb {R}^d$ we define $\textrm{dist}(x,E):=\inf _{e\in E}|x{-}e|$. We denote by $\mathcal {H}^{d-1}$ the $(d{-}1)$-dimensional Hausdorff measure and $x{\wedge }y{:}{=}\min \{x;y\}$ for all $x\,,y\in \mathbb {R}$.

In the following, we use the symbol $\dot{u}$ for the partial time derivative of the generic time-dependent function *u*, whereas $\frac{\textrm{d}}{\textrm{d} t}$ stands for the total time derivative, in case *u* depends on time only.

Henceforth, we indicate by *c* a generic positive constant possibly depending on data but independent of the parameter $\varepsilon$ and of the discretization step $\tau$. Note that the value of *c* may change even within the same line.

### Admissible deformations

Fix the final time $T>0$ and let the reference configuration $U \subset \mathbb {R}^d \ (d\ge 2)$ be nonempty, open, connected, and bounded. We assume that the boundary $\partial U$ is Lipschitz, with $\Gamma _D, \, \Gamma _N\subset \partial U$ disjoint and open in the topology of $\partial U$, $\Gamma _D \not = \emptyset$ and $\overline{\Gamma _D}\cup \overline{\Gamma _N} = \partial U$, where the closure is taken in the topology of $\partial U$. In the following, we use the short-hand notation $Q:=(0,T)\times U$ and $\Sigma _D:=(0,T)\times \Gamma _D$.

Deformations are assumed to belong to the affine space

$$\begin{aligned} W^{2,p}_{\Gamma _D}(U ;\mathbb {R}^d):=\left\{ y \in W^{2, p}(U ;\mathbb {R}^d)\,|\, y=\textrm{id} \text { on } \Gamma _D \right\} , \end{aligned}$$

for almost all times and some given

$$p>d.$$

Moreover, we impose local invertibility and orientation preservation. The set of *admissible deformations* is hence defined as

$$\begin{aligned} {{\mathcal {A}}} {:}{=}\left\{ y \in W^{2,p}_{\Gamma _D}(U;\mathbb {R}^d) \ \Big | \ \nabla y \in \textrm{GL}_+(d) \ \text {a.e. in } U\right\} . \end{aligned}$$

### Elastic energy

Let $\varepsilon \ge 0$ be given and $h_\varepsilon \in C^{\infty }(\mathbb {R};[0, 1])$ for $\varepsilon >0$ be nondecreasing functions such that

2.1 $$\begin{aligned} h_\varepsilon ( \sigma )={\left\{ \begin{array}{ll} 0 \quad &  \text {if} \ \ \sigma \le -\varepsilon /2,\\ 1 \quad &  \text {if} \ \ \sigma \ge \varepsilon /2, \end{array}\right. } \quad \Vert h_\varepsilon '\Vert _{L^{\infty }(\mathbb {R})}\le \frac{2}{\varepsilon }. \end{aligned}$$

Moreover, let $$ h_0$$ be the discontinuous Heaviside-like function defined as $$h_0( \sigma )=0$$ if $$ \sigma < 0$$ and $$h_0( \sigma )=1$$ if $$ \sigma \ge 0$$. Note that $$h_\varepsilon \rightarrow h_0$$ in $$\mathbb {R}\setminus \{0\}$$ as $$\varepsilon \rightarrow 0$$.

We define the elastic energy density $W_\varepsilon :\mathbb {R}\times \textrm{GL}_+(d)\rightarrow [0,\infty )$ of the medium as

2.2 $$\begin{aligned} W_\varepsilon (\sigma ,F){:}{=}(1-h_\varepsilon (\sigma )) V^a (F) + h_\varepsilon (\sigma ) V^{ r} (F) + V^{J}(F) . \end{aligned}$$

Here, $$\sigma $$ is a placeholder for $$\theta (x){-}t$$, whose 0-sublevel set $$\{x\in U\mid \theta (x)<t\}$$ represents the accreting phase at time $$t>0$$. In particular, $$W_\varepsilon (\sigma , \cdot )= V^a + V^{J}$$ for $$\sigma <-\varepsilon /2$$, so that $$ V^a + V^{J}$$ is the elastic energy density of the accreting phase. On the other hand, $$W_\varepsilon (\sigma ,\cdot )= V^r + V^{J}$$ for $$\sigma >\varepsilon /2$$ and $$ V^r + V^{J}$$ is the elastic energy density of the receding phase.

On the elastic energy densities we require

2.3 $$\begin{aligned}&V^a ,\, V^r ,\, V^{J}\in C^1(\textrm{GL}_+(d);[0,\infty )), \end{aligned}$$

2.4 $$\begin{aligned}&\exists c_W>0\,: \quad V^a(F),\, V^r(F)\ge c_W|F|^{p}-\frac{1}{c_W}, \nonumber \\&\quad V^a(F) - V^r(F) \le \frac{1}{c_W}(1+|F|^{p})\quad \forall F\in \textrm{GL}_+(d) , \end{aligned}$$

2.5 $$\begin{aligned}&\exists q>\frac{pd}{p-d}\,: \quad V^{J}(F)\ge \frac{c_W}{(\det F)^q}. \end{aligned}$$

The upper bound on $$V^a - V^r $$ in (2.4) will be instrumental in order to prove a control on the power associated with the phase transformation. In particular, if the receding phase has a higher energy density, namely, $$V^r \ge V^a$$, such upper bound trivially holds.

Although not strictly needed for the analysis we also require the frame-indifference

2.6 $$\begin{aligned} V^a (Q F)= V^a (F),\, V^r (Q F)= V^r (F), \, V^{J}(QF)=V^{J}(F) \ \ \forall F \in \textrm{GL}_+(d), \ Q \in \textrm{SO}(d). \end{aligned}$$

As regards the second-order potential *H* we ask for

2.7 $$\begin{aligned}&H \in C^1(\mathbb {R}^{d\times d \times d};[0,\infty ))\ \ \text {convex}, \end{aligned}$$

2.8 $$\begin{aligned}&H (Q G)=H (G) \ \ \text {for all} \ \ G \in \mathbb {R}^{d\times d \times d}, \ Q \in \textrm{SO}(d), \end{aligned}$$

2.9 $$\begin{aligned}&\exists c_H>0: \quad c_H|G|^p\le H(G) \le \frac{1}{c_H}(1+|G|)^p,\quad | \textrm{D} H(G)|\le \frac{1}{c_H}|G|^{p-1}, \end{aligned}$$

2.10 $$\begin{aligned}&c_H| G-\widehat{G}|^p\le ( \textrm{D} H( G)- \textrm{D} H( \widehat{G} )){\vdots }( G-\widehat{G})\quad \forall G,\, \widehat{G}\in \mathbb {R}^{d \times d \times d} . \end{aligned}$$

Again, the frame-indifference requirement (2.8) is not strictly needed for the analysis.

By integrating over the reference configuration *U* we define $\mathcal {W}_\varepsilon :C( \overline{U})\times {{\mathcal {A}}}\rightarrow [0,\infty )$ and $\mathcal {H}:\mathcal {A}\rightarrow [0,\infty )$ as

$$\begin{aligned} \mathcal {W}_\varepsilon (\sigma ,y){:}{=}\int \limits _{U }W_\varepsilon (\sigma ,\nabla y)\,\textrm{d} x\quad \text {and} \quad \mathcal {H}(y){:}{=}\int \limits _{U }H(\nabla ^2 y)\,\textrm{d} x. \end{aligned}$$

### Viscous dissipation

For $\varepsilon \ge 0$ given, set the instantaneous viscous dissipation density $R_\varepsilon :\mathbb {R}\times \textrm{GL}_+(d) \times \mathbb {R}^{d\times d}\rightarrow [0,\infty )$ as

2.11 $$\begin{aligned} R_\varepsilon (\sigma ,F,\dot{F}){:}{=}(1-h_\varepsilon (\sigma ))R^a(F,\dot{F}) + h_\varepsilon (\sigma ) R^{ r}(F,\dot{F}) \end{aligned}$$

Here, $R^a,\, R^{ r}:\textrm{GL}_+(d) \times \mathbb {R}^{d\times d}\rightarrow [0,\infty )$ are the instantaneous viscous dissipation densities of the accreting and of the receding phase, respectively. They are assumed to be quadratic in the rate $\dot{C}{:}{=}\dot{F}^\top F+ F^\top \dot{F}$ of the right Cauchy–Green tensor $C{:}{=}F^\top F$, namely

$$\begin{aligned} R^a(F,\dot{F}){:}{=}\frac{1}{2}\dot{C} {:}\mathbb {D}^a(C) {:}\dot{C}, \quad R^{ r}(F,\dot{F}){:}{=}\frac{1}{2}\dot{C} {:}\mathbb {D}^{ r}(C) {:}\dot{C} \qquad \forall F \in \textrm{GL}_+(d), \ \dot{F}\in \mathbb {R}^{d\times d}. \end{aligned}$$

We assume that

2.12 $$\begin{aligned}&\mathbb {D}^a,\, \mathbb {D}^{ r}\in C(\mathbb {R}^{d\times d}_\textrm{sym};\mathbb {R}^{d\times d\times d\times d}) \ \ \text {with} \ \ (\mathbb {D}^i)_{jk\ell m}=(\mathbb {D}^i)_{kj \ell m}=(\mathbb {D}^i)_{\ell mjk} \nonumber \\&\quad \forall j,\,k,\,\ell ,\,m=1,\dots , d, \ \text {for} \ i=a,\, r , \end{aligned}$$

2.13 $$\begin{aligned}&\exists c_{\mathbb {D}}>0: \quad c_{\mathbb {D}}|\dot{C}|^2\le \dot{C} {:}\mathbb {D}^{ i}(C) {:}\dot{C} \quad \forall C,\,\dot{C}\in \mathbb {R}^{d\times d}_\textrm{sym}, \ \text {for} \ i=a,\, r. \end{aligned}$$

Notice that this specific choice of $R_\varepsilon$ ensures that

$$\begin{aligned}&\partial _{\dot{F}}R_\varepsilon (\sigma ,F,\dot{F})= 2(1{-}h_\varepsilon (\sigma ))F\mathbb {D}^a(C) {:}\dot{C} +2h_\varepsilon (\sigma )F\mathbb {D}^{ r}(C) {:}\dot{C}\\&\quad = 2(1{-}h_\varepsilon (\sigma ))F\mathbb {D}^a(F^\top F) {:} (\dot{F}^\top F{+} F^\top \dot{F}) + 2h_\varepsilon (\sigma )F\mathbb {D}^{ r} (F^\top F){:}(\dot{F}^\top F{+} F^\top \dot{F}), \end{aligned}$$

which is of course linear in $\dot{F}$. By integrating on the reference configuration *U* we define $\mathcal {R}_\varepsilon :C(\overline{U})\times \mathcal {A} \times H^{1}(U;\mathbb {R}^d)\rightarrow [0,\infty )$ as

$$\begin{aligned} \mathcal {R}_\varepsilon (\sigma ,y,\dot{y}){:}{=}\int \limits _U R_\varepsilon (\sigma ,\nabla y,\nabla \dot{y})\,\textrm{d} x. \end{aligned}$$

### Loading and initial data

We assume that the body force density $f=f(\sigma ,x)$ is (constant in time and) suitably smooth with respect to $\sigma$, namely

2.14 $$\begin{aligned} f \in W^{1, \infty }(\mathbb {R};L^{2}(U ;\mathbb {R}^d)), \end{aligned}$$

The $\sigma$-dependence of the force density *f* is intended to cover the case of gravitation $f=\rho g$, where the density $\rho$ depends on the phase, while the acceleration field *g* is given.

We moreover assume that the initial deformation $y_0$ satisfies

2.15 $$\begin{aligned}&y_0\in \mathcal {A} \ \ \text {with} \ \int \limits _U V^a (\nabla y_0 )+ V^r (\nabla y_0 )+V^{J}(\nabla y_0)+H(\nabla ^2 y_0)\,\textrm{d} x<\infty . \end{aligned}$$

### Growth

Concerning the accretive-growth model we ask for

2.16 $$\begin{aligned} \gamma \in C^{0,1}(\mathbb {R}^d \times \textrm{GL}_+(d)) \ \ \text {with} \ \ c_\gamma \le \gamma (\cdot ) \le C_\gamma \ \ \text {for some} \ \ 0<c_\gamma \le C_\gamma . \end{aligned}$$

Let moreover the initial location of the accreting phase be given by

2.17 $$\begin{aligned} \emptyset \not =\Omega _0 \subset \subset U\ \ \text {with} \ \ \Omega _0 \ \ \text {open and} \ \ \Omega _0+B_{C_\gamma T}\subset \subset U. \end{aligned}$$

As it will be clarified later, this last requirement guarantees that the accreting phase does not touch the boundary $\partial U$ over the time interval [0, *T*], see (4.25).

### Main results

Assumptions (2.1)–(2.17) will be assumed throughout the remainder of the paper. We are interested in solving (1.1)–(1.7) in the following weak/viscosity sense.

#### Definition 2.1

**(Weak/viscosity solution)** We say that a pair

$$\begin{aligned} (y, \theta ) \in \left( L^{\infty }(0,T; W^{2,p}(U;\mathbb {R}^d ))\cap H^1(0,T;H^1(U;\mathbb {R}^d))\right) \times C^{0,1}({\overline{U}}) \end{aligned}$$

is a *weak/viscosity solution* to the initial-boundary-value problem (1.1)–(1.7) if $y(t,\cdot ) \in {\mathcal {A}}$ for all $t\in (0,T)$, $y(0,\cdot )=y_0$, and

2.18 $$\begin{aligned}&\int \limits _0^T\!\! \int \limits _{U } \big (\partial _F W_\varepsilon (\theta {-}t,\nabla y){:}\nabla z{+}\partial _{\dot{F}} R_\varepsilon (\theta {-}t,\nabla y, \nabla \dot{y}){:}\nabla z +\textrm{D}H\left( \nabla ^2 y\right) {\vdots } \nabla ^2 z \big )\,\textrm{d} x\,\textrm{d} t\nonumber \\&\quad = \int \limits _0^T \!\!\int \limits _{U } f(\theta {-}t) {\cdot }z \,\textrm{d} x\,\textrm{d} t\quad \forall z\in C^{\infty }( \overline{Q }; \mathbb {R}^d) \ \text {with} \ z=0 \ \text {on} \ \Sigma _D, \end{aligned}$$

and $\theta$ is a viscosity solution to

2.19 $$\begin{aligned}&\gamma \big (y(\theta (x) \wedge T,x),\nabla y(\theta (x) \wedge T,x)\big )|\nabla (-\theta )(x)|=1 \ \ \text {in} \ \ U \setminus \overline{\Omega _0}, \end{aligned}$$

2.20 $$\begin{aligned}&\theta =0 \ \ \text {in} \ \ \Omega _0. \end{aligned}$$

Namely, $\theta$ satisfies (2.20), and, for all $x_0 \in U\setminus \overline{\Omega _0}$ and any smooth function $\varphi :U \rightarrow \mathbb {R}$ with $\varphi (x_0)=-\theta (x_0)$ and $\varphi \ge -\theta$ ($\varphi \le -\theta$, respectively) in a neighborhood of $x_0$, it holds that $\gamma (y(\theta (x_0) \wedge T,x_0), \nabla y(\theta (x_0) \wedge T,x_0))|\nabla \varphi (x_0))|\le 1 (\ge 1, \ \text {respectively})$. Moreover, we ask that

2.21 $$\begin{aligned} 0<\frac{1}{C_\gamma } \le |\nabla \theta | \le \frac{1}{c_\gamma } \ \ \text {a.e. in} \ \ U. \end{aligned}$$

Note that this weak notion of solution in Definition 2.1 still entails the validity of an energy equality. Namely, we have the following.

#### Proposition 2.1

(Energy equality) Under assumptions (2.1)–(2.17), in the diffused-interface case $\varepsilon >0$ a weak/viscosity solution $(y,\theta )$ fulfills for all $t\in [0,T]$ the energy equality

2.22 $$\begin{aligned}&\int \limits _U \big (W_\varepsilon (\theta {-}t,\nabla y )+H(\nabla ^2 y)- f(\theta {-}t){\cdot }y\big ) \,\textrm{d} x- \int \limits _U \left( W_\varepsilon (\theta ,\nabla y_0 )+H(\nabla ^2 y_0)- f(\theta ){\cdot }y_0\right) \,\textrm{d} x\nonumber \\&\quad = -2\int \limits _0^{t}\!\!\int \limits _U R_\varepsilon \left( \theta {-}s,\nabla y,\nabla \dot{y}\right) \,\textrm{d} x\,\textrm{d} s-\int \limits _{0}^{t}\!\!\int \limits _U \dot{f}(\theta {-}s){\cdot } y\,\textrm{d} x\,\textrm{d} s\nonumber \\&\qquad -\int \limits _0^{t}\!\!\int \limits _U \partial _\sigma W_\varepsilon (\theta {-}s,\nabla y )\,\textrm{d} x\,\textrm{d} s. \end{aligned}$$

In the sharp-interface case $\varepsilon =0$, for all $t\in [0,T]$, one has instead

2.23 $$\begin{aligned}&\int \limits _U \big (W_0 (\theta {-}t,\nabla y )+H(\nabla ^2 y)- f(\theta {-}t){\cdot }y\big ) \,\textrm{d} x- \int \limits _U \left( W_0 (\theta ,\nabla y_0 )+H(\nabla ^2 y_0)- f(\theta ){\cdot }y_0\right) \,\textrm{d} x\nonumber \\&\quad = -2\int \limits _0^{t}\!\!\int \limits _U R_0\left( \theta {-}s,\nabla y,\nabla \dot{y}\right) \,\textrm{d} x\,\textrm{d} s-\int \limits _{0}^{t}\!\!\int \limits _U \dot{f}(\theta {-}s){\cdot } y\,\textrm{d} x\,\textrm{d} s\nonumber \\&\qquad -\int \limits _0^{t}\!\!\int \limits _{\{\theta =s\}} \frac{ V^r (\nabla y )- V^a (\nabla y)}{|\nabla \theta |}\,\textrm{d}\mathcal {H}^{d-1}\,\textrm{d} s. \end{aligned}$$

Relations (2.22)–(2.23) express the energy balance in the model. In particular, the difference between the actual and the initial complementary energies (left-hand side in (2.22)–(2.23)) equals the sum of the total viscous dissipation, the work of external forces, and the energy stored in the medium in connection with the phase-transition process (the three terms in the right-hand side of (2.22)–(2.23), up to signs). Proposition 2.1 is proved in Sect. 3.

Our main result reads as follows.

#### Theorem 2.1

(Existence) Under assumptions (2.1)–(2.17), for all given $\varepsilon \ge 0$ there exists a weak/viscosity solution $(y, \theta )$ of problem (1.1)–(1.7).

A proof of Theorem 2.1 in the diffused-interface case of $\varepsilon >0$ is based on an iterative strategy: for given $y^k$ one finds a viscosity solution $\theta ^k$ to (2.19)–(2.20) (with *y* replaced by $y^k$). Then, given $\theta ^k$ one can find $y^{k+1}$ satisfying (2.18) (with $\theta$ replaced by $\theta ^k$). Note that such $y^{k+1}$ may be nonunique. As the set of solutions *y* for given $\theta$ is generally not convex, we do not proceed via a fixed-point argument for multivalued maps (see, e.g., [24]) but rather resort in directly proving the convergence of the iterative procedure. This argument is detailed in Sect. 4.

Eventually, the proof of Theorem 2.1 in the sharp-interface case $\varepsilon =0$ will be obtained in Sect. 5 by passing to the limit as $\varepsilon \rightarrow 0$ along a subsequence of weak/viscosity solutions $(y_\varepsilon ,\theta _\varepsilon )$ for $\varepsilon >0$. As a by-product, we have the following.

#### Corollary 2.1

(Sharp-interface limit) Under assumptions (2.1)–(2.17), let $(y_\varepsilon ,\theta _\varepsilon )$ be weak/viscosity solutions of the diffused-interface problem (1.1)–(1.7) for $\varepsilon >0$. Then, there exists a not relabeled subsequence such that $(y_\varepsilon ,\theta _\varepsilon )\rightarrow (y,\theta )$ uniformly, where $(y,\theta )$ is a weak/viscosity solution to the sharp-interface problem for $\varepsilon =0$.

Before moving on, let us mention that the assumptions on the energy and of the instantaneous viscous dissipation density could be generalized by not requiring the specific forms (2.2) and (2.11). In fact, one could directly assume to be given $W_\varepsilon =W_\varepsilon (\sigma ,F)$ and $R_\varepsilon =R_\varepsilon (\sigma ,F,\dot{F})$ of the form

$$\begin{aligned} R_\varepsilon (\sigma ,F,\dot{F}) = \frac{1}{2} \dot{C} {:}{{\mathbb {D}}}(\sigma ,C){:} \dot{C} \end{aligned}$$

with ${{\mathbb {D}}}\in C(\mathbb {R}\times \mathbb {R}_\textrm{sym}^{d\times d};\mathbb {R}^{d \times d \times d \times d})$ by suitably adapting the smoothness and coercivity assumptions. Although the existence analysis could be carried out in this more general situation with no difficulties, we prefer to stick to the concrete choice of (2.2) and (2.11) as it allows a more transparent distinction of the diffused- and sharp-interface cases.

Moreover, let us point out that admissible deformations *y* are presently required to be solely *locally* injective, by means of the constraint $\det \nabla y >0$. On the other hand, *global* injectivity may also be enforced, in the spirit of [25], see also [42] in the static and [9, 10] in the dynamic case. This however calls for keeping track of reaction forces due to a possible self-contact at the boundary $\Gamma _N$. From the technical viewpoint, one would need to include an extra variable in the state in order to model such reaction. The existence theory of Theorem 2.1 can be extended to cover this case, at the price of some notational intricacies. We however prefer to avoid discussing global injectivity here, for the sake of exposition clarity.

## Proof of Proposition 2.1: energy equalities

We firstly consider the diffused-interface setting of $\varepsilon >0$. Let $(y,\theta )$ be a weak/viscosity solution to (1.1)–(1.7). In order to deduce the energy equality, the Euler-Lagrange equation (2.18) should be tested by $\dot{y}$. This however requires some care, as $\dot{y}$ is not regular enough to use it as test function. We follow the argument of [36], based on the validity of a chain rule for the functional $\mathcal {H}$. In particular, we start by checking that (2.18) can be equivalently rewritten as

3.1 $$\begin{aligned} \partial _2\mathcal {W}_\varepsilon (\theta {-}t,y) + \partial _3\mathcal {R}_\varepsilon (\theta {-}t,y,\dot{y}) +\partial \mathcal {H}(y) \ni \widehat{f} \quad \text {in} \ \ (H^1_{\Gamma _D}(U;\mathbb {R}^{ d}))^*, \ \ \text {a.e. in} \ \ (0,T). \end{aligned}$$

Here, $(H^1_{\Gamma _D}(U;\mathbb {R}^{ d}))^*$ indicates the dual of $H^1_{\Gamma _D} (U;\mathbb {R}^{ d}):=\{z \in H^1 (U;\mathbb {R}^{d}) \ | \ z=0 \ \text {on} \ \Gamma _D\}$, the symbol $\partial$ denotes the (possibly partial) subdifferential from $H^1_{\Gamma _D} (U;\mathbb {R}^{ d})$ to $(H^1_{\Gamma _D}(U;\mathbb {R}^{ d}))^*$ and $\widehat{f}: (0,T)\rightarrow (H^1_{\Gamma _D}(U;\mathbb {R}^{ d}))^*$ is given by

$$\begin{aligned} \langle \widehat{f}(t),z\rangle := \int \limits _U f(\sigma {-}t){\cdot } z \, \textrm{d} x\quad \forall z \in H^1_{\Gamma _D} (U;\mathbb {R}^{ d}) \end{aligned}$$

where $\langle \cdot ,\cdot \rangle$ is the duality pairing between $(H^1_{\Gamma _D}(U;\mathbb {R}^{d}))^*$ and $H^1_{\Gamma _D}(U;\mathbb {R}^{ d})$. Indeed, owing to the fact that $\nabla y \in L^\infty (Q)$ and $\nabla \dot{y} \in L^2(Q)$ and using the regularities (2.3), (2.12), and (2.14) one can check that

$$\begin{aligned}&\langle \partial _2\mathcal {W}_\varepsilon (\theta {-}t,y) ,z\rangle = \int \limits _U \partial _FW_\varepsilon (\theta {-}t,\nabla y){:}\nabla z \quad \forall z \in H^1_{\Gamma _D} (U;\mathbb {R}^{ d}),\\&\langle \partial _3\mathcal {R}_\varepsilon (\theta {-}t,y,\dot{y}) ,z\rangle = \int \limits _U \partial _{\dot{F}}R_\varepsilon (\theta {-}t,\nabla y,\nabla \dot{y}){:}\nabla z \quad \forall z \in H^1_{\Gamma _D} (U;\mathbb {R}^{ d}), \end{aligned}$$

and that $\Sigma = \widehat{f} - \partial _2\mathcal {W}_\varepsilon (\theta {-}t,y) - \partial _3\mathcal {R}_\varepsilon (\theta {-}t,y,\dot{y}) \in L^2(0,T; (H^1_{\Gamma _D}(U;\mathbb {R}^{d}))^*)$. On the other hand, using equation (2.18), the fact that $y\in L^p(0,T;W^{2,p}(\Omega ;\mathbb {R}^d))$, and the convexity (2.7) of *H* we get

$$\begin{aligned} \int \limits _0^T \langle \Sigma , w-y\rangle \, \textrm{d}t {\mathop {=}\limits ^{(2.18)}} \int \limits _0^T \!\!\int \limits _U\textrm{D}H(\nabla ^2y){\vdots }\nabla ^2(w-y)\, \textrm{d}t \le \int \limits _0^T \big (\mathcal {H}(w)-\mathcal {H}(y)\big )\, \textrm{d}t \end{aligned}$$

for all $w\in L^p(0,T;W^{2,p}(\Omega ;\mathbb {R}^d))\cap L^2(0,T; H^1_{\Gamma _D}(U;\mathbb {R}^{d}))$. This in particular implies that $\Sigma \in \partial \mathcal {H}(y)$ a.e. in (0, *T*), whence the abstract equation (3.1) follows and the chain rule [36, Prop. 3.6] entails that $\mathcal {H}(y)\in W^{1,1}(0,T)$ and

3.2 $$\begin{aligned} \frac{\textrm{d}}{\textrm{d}t} \mathcal {H}(y) = \langle \Sigma ,\dot{y} \rangle \quad \text {a.e. in} \ (0,T). \end{aligned}$$

Note that all terms in (3.1) belong to $L^2(0,T; (H^1_{\Gamma _D}(U;\mathbb {R}^{d}))^*)$. One can hence test (3.1) on $\dot{y} \in L^2(0,T; H^1_{\Gamma _D}(U;\mathbb {R}^{d}))$ and deduce that

3.3 $$\begin{aligned}&\int \limits _0^t \!\!\int \limits _U \partial _F W_\varepsilon (\theta {-}s,\nabla y){:}\nabla \dot{y} \,\textrm{d} x\, \textrm{d} s+ \int \limits _0^t \!\!\int \limits _U \partial _{\dot{F}}R_\varepsilon (\theta {-}s,\nabla y , \nabla \dot{y}){:} \nabla \dot{y} \, \textrm{d} x\, \textrm{d} s\nonumber \\&\quad +\int \limits _U H(\nabla ^2y(t))\, \textrm{d} x- \int \limits _U H(\nabla ^2y_0)\, \textrm{d} x= \int \limits _0^t \!\!\int \limits _U f(\theta {-}s){\cdot } \dot{y}\, \textrm{d} x\,\textrm{d} s. \end{aligned}$$

We readily have that

3.4 $$\begin{aligned}&\int \limits _U W_\varepsilon (\theta {-}t,\nabla y)\, \textrm{d} x-\int \limits _U W_\varepsilon (\theta ,\nabla y_0)\, \textrm{d} x=\int \limits _0^t\frac{\textrm{d}}{ \textrm{d}s } \int \limits _U \partial _F W_\varepsilon (\theta {-}s,\nabla y)\, \textrm{d} x\, \textrm{d} s\nonumber \\&\quad =\int \limits _0^t \!\!\int \limits _U \partial _F W_\varepsilon (\theta {-}s,\nabla y){:}\nabla \dot{y} \,\textrm{d} x\, \textrm{d} s- \int \limits _0^t \!\!\int \limits _U \partial _\sigma W_\varepsilon (\theta {-}s,\nabla y)\, \textrm{d} x\, \textrm{d} s. \end{aligned}$$

Moreover, it is a standard matter to check that $\partial _{\dot{F}}R_\varepsilon (\sigma ,F,\dot{F}){:}\dot{F} = 2 R_\varepsilon (\sigma ,F,\dot{F})$, so that

3.5 $$\begin{aligned} \int \limits _0^t \!\!\int \limits _U \partial _{\dot{F}}R_\varepsilon (\theta {-}s,\nabla y , \nabla \dot{y}){:} \nabla \dot{y} \, \textrm{d} x\, \textrm{d} s= 2\int \limits _0^t \!\!\int \limits _U R_\varepsilon (\theta {-}s,\nabla y , \nabla \dot{y})\, \textrm{d} x\, \textrm{d} s, \end{aligned}$$

whence the energy equality (2.22) in the diffused-interface case $\varepsilon >0$ follows from (3.3).

The proof of energy equality (2.23) for the sharp-interface case $\varepsilon =0$ follows the same strategy, as one can again establish (3.3) (for $W_0$ and $R_0$ in place of $W_\varepsilon$ and $R_\varepsilon$) and (3.2). A notable difference is however in (3.4), which now requires some extra care as $h_0$ is discontinuous. In particular, the energy equality (2.23) follows as soon as we prove that

3.6 $$\begin{aligned}&\int \limits _U W_0(\theta {-}t,\nabla y)\, \textrm{d} x-\int \limits _U W_0(\theta ,\nabla y_0)\, \textrm{d} x\nonumber \\&\quad =\int \limits _0^t \!\!\int \limits _U \partial _F W_0(\theta {-}s,\nabla y){:}\nabla \dot{y} \,\textrm{d} x\, \textrm{d} s- \int \limits _0^t \!\!\int \limits _{\{\theta =s\}} \frac{ V^r (\nabla y) - V^a (\nabla y)}{|\nabla \theta |} \, \textrm{d} \mathcal {H}^{d-1} \, \textrm{d} s. \end{aligned}$$

The remainder of this section is devoted to check (3.6).

To start with, let a nonnegative and even function $\rho \in C^\infty (\mathbb {R})$ be given with support in $[-1,1]$ and with $\int \limits _\mathbb {R}\rho (s)\, \textrm{d} s=1$. For $\varepsilon >0$ we define $\rho _\varepsilon (t):= \rho (t/\varepsilon )/\varepsilon$ and $\eta _\varepsilon (t):= \int \limits _{-1}^t\rho _\varepsilon (s)\, \textrm{d} s$ for all $t \in \mathbb {R}$. As $\eta _\varepsilon \rightarrow h_0$ in $\mathbb {R}\setminus \{0\}$, by letting

$$\begin{aligned} G_\varepsilon (t):= \int \limits _U \Big ( V^a (\nabla y(t,x)) + \eta _\varepsilon (\theta (x){-}t) \big ( V^r (\nabla y(t,x)) - V^a (\nabla y(t,x))\big )+V^{J}(\nabla y(t,x)) \Big ) \, \textrm{d} x\end{aligned}$$

we readily check that

3.7 $$\begin{aligned} \lim _{\varepsilon \rightarrow 0}G_\varepsilon (t):=\int \limits _U W_0(\theta (x){-}t,\nabla y(t,x))\, \textrm{d} x \end{aligned}$$

for all $t \in [0,T]$. As $G_\varepsilon \in H^1(0,T)$ we can compute its time derivative at almost all times getting

$$\begin{aligned} \frac{\textrm{d}}{\textrm{d} t} G_\varepsilon (t)&= \int \limits _U \Big ( \partial _F V^a (\nabla y)+ \eta _\varepsilon (\theta {-}t) \big ( \partial _F V^r (\nabla y) {-}\partial _F V^a (\nabla y)\big ) +\partial _F V^{J}(\nabla y)\Big ) {:}\nabla \dot{y} \, \textrm{d} x\\&\quad  -\int \limits _U\rho _\varepsilon (\theta {-}t) \big ( V^r (\nabla y) {-} V^a (\nabla y)\big ) \, \textrm{d} x. \end{aligned}$$

By integrating in time, taking the limit $\varepsilon \rightarrow 0$, and using (3.7), one hence gets

$$\begin{aligned}&\int \limits _U W_0(\theta {-}t,\nabla y)\, \textrm{d} x- \int \limits _U W_0(\theta ,\nabla y_0)\, \textrm{d} x= \lim _{\varepsilon \rightarrow 0} \big (G_\varepsilon (t) - G_\varepsilon (0) \big )= \lim _{\varepsilon \rightarrow 0}\int \limits _0^t \frac{\textrm{d}}{\textrm{d}s} G_\varepsilon (s)\, \textrm{d} s\\&\quad = \lim _{\varepsilon \rightarrow 0}\int \limits _0^t\!\!\int \limits _U \Big ( \partial _F V^a(\nabla y)+ \eta _\varepsilon (\theta {-}s) \big ( \partial _F V^r (\nabla y) {-}\partial _F V^a (\nabla y)\big ) +\partial _F V^{J}(\nabla y)\Big ) {:}\nabla \dot{y} \, \textrm{d} x\, \textrm{d} s\\&\qquad  -\lim _{\varepsilon \rightarrow 0}\int \limits _0^t\!\! \int \limits _U\rho _\varepsilon (\theta {-}s) \big ( V^r (\nabla y) {-} V^a (\nabla y)\big ) \, \textrm{d} x\, \textrm{d} s\\&\quad = \int \limits _0^t \!\!\int \limits _U \partial _F W_0(\theta {-}s,\nabla y){:}\nabla \dot{y} \,\textrm{d} x\, \textrm{d} s-\lim _{\varepsilon \rightarrow 0}\int \limits _0^t\!\! \int \limits _U\rho _\varepsilon (\theta {-}s) \big ( V^r (\nabla y) {-} V^a (\nabla y)\big ) \, \textrm{d} x\, \textrm{d} s. \end{aligned}$$

In order to prove (3.6) it is hence sufficient to check that

3.8 $$\begin{aligned}&\lim _{\varepsilon \rightarrow 0}\int \limits _0^t\!\! \int \limits _U\rho _\varepsilon (\theta {-}s) \big ( V^r (\nabla y) {-} V^a (\nabla y)\big ) \, \textrm{d} x\, \textrm{d} s=\int \limits _0^t \!\!\int \limits _{\{\theta =s\}} \frac{ V^r (\nabla y) {-} V^a (\nabla y)}{|\nabla \theta |} \, \textrm{d} \mathcal {H}^{d-1} \, \textrm{d} s. \end{aligned}$$

By introducing the short-hand notation $g = V^r (\nabla y) - V^a (\nabla y)$ and by using the coarea formula [16, Sec. 3.2.11] (recall that $\theta$ is Lipschitz continuous and $|\nabla \theta | \ge 1/C_\gamma >0$ almost everywhere, see (2.21)) we can compute

3.9 $$\begin{aligned}&\int \limits _0^t\!\! \int \limits _U\rho _\varepsilon (\theta {-}s) \big ( V^r (\nabla y) {-} V^a (\nabla y)\big ) \, \textrm{d} x\, \textrm{d} s= \int \limits _0^t\!\!\int \limits _\mathbb {R}\int \limits _{\{\theta =r\}} \rho _\varepsilon (\theta (x) {-}s)\frac{g(s,x)}{|\nabla \theta (x)|} \textrm{d}\mathcal {H}^{d-1}(x)\, \textrm{d}r \, \textrm{d} s\nonumber \\&\quad = \int \limits _0^t\!\!\int \limits _\mathbb {R}\int \limits _{\{\theta =r\}}\rho _\varepsilon (r{-}s) \frac{g(r,x)}{|\nabla \theta (x)|} \textrm{d}\mathcal {H}^{d-1}(x)\, \textrm{d}r \, \textrm{d} s\nonumber \\&\qquad + \int \limits _0^t\!\!\int \limits _\mathbb {R}\int \limits _{\{\theta =r\}}\rho _\varepsilon (r{-}s) \frac{g(s,x){-}g(r,x)}{|\nabla \theta (x)|} \textrm{d}\mathcal {H}^{d-1}(x)\, \ \textrm{d}r \, \textrm{d} s. \end{aligned}$$

The coarea formula and the bound $|\nabla \theta |\le 1/c_\gamma$ (see again (2.21)) ensure that $r\in \mathbb {R}\mapsto m (r):= \mathcal {H}^{d-1}(\{\theta = r\})$ is integrable. Indeed,

$$\begin{aligned} \Vert m \Vert _{L^1(\mathbb {R})}=\int \limits _\mathbb {R}\mathcal {H}^{d-1}(\{\theta = r\}) \, \textrm{d} r= \int \limits _U |\nabla \theta |\,\textrm{d} x<\infty . \end{aligned}$$

As $g/|\nabla \theta |$ is bounded, setting

$$\begin{aligned} r\in \mathbb {R}\mapsto \ell (r):= \int \limits _{\{\theta =r\}} \frac{g(r,x)}{|\nabla \theta (x)|} \textrm{d}\mathcal {H}^{d-1}(x) \end{aligned}$$

one has that $\ell \in L^1(\mathbb {R})$, as well, since

$$\begin{aligned} \Vert \ell \Vert _{L^1(\mathbb {R})} = \int \limits _\mathbb {R}\!\! \int \limits _{\theta = r} \frac{|g(r,x)|}{|\nabla \theta (x)|}\,\textrm{d}\mathcal {H}^{d-1}(x)\, \textrm{d}r \le \sup \frac{|g|}{|\nabla \theta |} \Vert m \Vert _{L^1(\mathbb {R})}<\infty . \end{aligned}$$

Moreover, we have that

$$\begin{aligned}&\int \limits _\mathbb {R}\int \limits _{\{\theta =r\}}\rho _\varepsilon (r{-}s) \frac{g(r,x)}{|\nabla \theta (x)|} \textrm{d}\mathcal {H}^{d-1}(x)\, \textrm{d}r \\&\quad = \int \limits _\mathbb {R}\rho _\varepsilon (s{-}r)\left( \int \limits _{\{\theta =r\}} \frac{g(r,x)}{|\nabla \theta (x)|} \textrm{d}\mathcal {H}^{d-1}(x)\right) \, \textrm{d}r = (\rho _\varepsilon *\ell )(s) \end{aligned}$$

where we used that $\rho _\varepsilon$ is even and where the symbol $*$ stands for the usual convolution in $\mathbb {R}$. As $\rho _\varepsilon *\ell \rightarrow \ell$ strongly in $L^1(\mathbb {R})$ for $\varepsilon \rightarrow 0$, one can pass to the limit in the first term on the right-hand side of (3.9) and get

3.10 $$\begin{aligned}&\lim _{\varepsilon \rightarrow 0}\int \limits _0^t\!\!\int \limits _\mathbb {R}\int \limits _{\{\theta =r\}}\rho _\varepsilon (r{-}s) \frac{g(r,x)}{|\nabla \theta (x)|} \textrm{d}\mathcal {H}^{d-1}(x)\, \textrm{d}r \, \textrm{d} s= \lim _{\varepsilon \rightarrow 0} \int \limits _0^t (\rho _\varepsilon *\ell )\,\textrm{d} s= \int \limits _0^t \ell \, \textrm{d} s\nonumber \\&\quad = \int \limits _0^t\!\!\int \limits _{\{\theta =s\}} \frac{g(s,x)}{|\nabla \theta (x)|} \,\textrm{d}\mathcal {H}^{d-1}(x)\, \textrm{d}s = \int \limits _0^t\!\!\int \limits _{\{\theta =s\}} \frac{ V^r (\nabla y){-} V^a (\nabla y)}{|\nabla \theta |}\, \textrm{d}\mathcal {H}^{d-1}\, \textrm{d}s. \end{aligned}$$

As regards the second term in the right-hand side of (3.9), notice that $\rho _\varepsilon (r{-}s)\ne 0$ only if $|r-s|\le 2\varepsilon$. Hence, using the Hölder regularity of *g* and the boundedness of $1/|\nabla \theta |$ and $|\nabla \theta |$, we conclude that

3.11 $$\begin{aligned}&\lim _{\varepsilon \rightarrow 0}\left| \int \limits _0^t\!\!\int \limits _\mathbb {R}\int \limits _{\{\theta =r\}}\rho _\varepsilon (r{-}s) \frac{g(s,x){-}g(r,x)}{|\nabla \theta (x)|} \textrm{d}\mathcal {H}^{d-1}(x)\, \textrm{d}r \, \textrm{d} s\right| \nonumber \\&\quad \le \lim _{\varepsilon \rightarrow 0} c \,\varepsilon ^\alpha \int \limits _0^t\!\!\int \limits _\mathbb {R}\rho _\varepsilon (r{-}s) \,\mathcal {H}^{d-1}(\{\theta =r\})\, \textrm{d}r \, \textrm{d} s\nonumber \\&\quad = \lim _{\varepsilon \rightarrow 0} c \, \varepsilon ^\alpha \Vert \rho _\varepsilon *m \Vert _{L^1(\mathbb {R})}\le \lim _{\varepsilon \rightarrow 0} c \, \varepsilon ^\alpha \Vert m\Vert _{L^1(\mathbb {R})} =0 \end{aligned}$$

for some $\alpha \in (0,1)$. Relations (3.10)–(3.11) imply that the limit (3.8) holds true. This in turn proves (3.6) and the energy equality (2.23) follows.

## Proof of Theorem 2.1: diffused-interface case

Let $\varepsilon >0$ be fixed. We prove existence of a weak/viscous solution $(y,\theta )$ by an iterative construction. We start by proving that for all given $\theta \in C( \overline{U} )$ there exists an admissible deformation *y* satisfying (2.18).

### Proposition 4.1

(Existence of *y* given $\theta$) Set $\varepsilon > 0$ and let $\theta \in C( \overline{U} )$ be fixed with $\Omega (T) \subset \subset U$. Under assumptions (2.1)–(2.15) there exists $y\in L^{\infty }(0,T;W^{2,p}(U;\mathbb {R}^d))\cap H^1(0,T;H^1(U;\mathbb {R}^d))$ with $y(t,\cdot ) \in \mathcal {A}$ for every $t\in (0,T)$ satisfying (2.18). More precisely, there exists a positive constant *c* depending on data but independent of $\varepsilon$ and $\theta$ such that

4.1 $$\begin{aligned} \Vert y\Vert _{L^{\infty }(0,T;W^{2,p}(U ;\mathbb {R}^d))\cap H^1(0,T;H^1(U ;\mathbb {R}^d))}\le c. \end{aligned}$$

### Proof

The assertion follows by adapting the arguments in [2] or [25]. We proceed by time discretization. Let the time step $\tau {:}{=}T/N_\tau$ with $N_\tau \in \mathbb {N}$ be given and let $t_i{:}{=}i \tau$, for $i=0,\dots , N_\tau$ be the corresponding uniform partition of the time interval [0, *T*]. Within this proof, the generic constant *c* is always independent of the given $\theta$, as well.

For $i=1,\dots , N_\tau$, we define $y^i_\tau \in \mathcal {A}$ via

$$\begin{aligned} y^i_\tau \in \mathop {\mathrm {arg\,min}}\limits _{ y\in {\mathcal {A}}}\left\{ \mathcal {W}_\varepsilon (\theta {-}t_i,y)+\mathcal {H}(y)+\tau \mathcal {R}_\varepsilon \left( \theta {-}t_i,y^{i-1}_\tau ,\frac{y{-}y^{i-1}_\tau }{\tau }\right) -\int \limits _U f(\theta {-}t_i){\cdot }y\,\textrm{d} x\right\} . \end{aligned}$$

Under the growth conditions (2.4)–(2.5), (2.9), and (2.13), and the regularity and convexity assumptions (2.3), (2.7), (2.12), and (2.14), the existence of $y^i_\tau$ for every $i=1,\dots , N_\tau$ follows by the Direct Method of the calculus of variations. Moreover, every minimizer $y_\tau ^i$ satisfies the time-discrete Euler–Lagrange equation

4.2 $$\begin{aligned}&\int \limits _{U } \left( \partial _F W_\varepsilon (\theta {-}t_i,\nabla y^i_\tau ){+}\partial _{\dot{F}} R_\varepsilon \left( \theta {-}t_i,\nabla y^{i-1}_\tau , \frac{\nabla y^i_\tau {-}\nabla y^{i-1}_\tau }{\tau }\right) \right) {:}\nabla z^i \,\textrm{d} x\nonumber \\&\quad +\int \limits _U { \mathrm D}H\left( \nabla ^2 y^i_\tau \right) {\vdots } \nabla ^2 z^i \,\textrm{d} x= \int \limits _U f(\theta {-}t_i){\cdot }z\,\textrm{d} x\end{aligned}$$

for every $$z^i \in \mathcal {A} $$.

Let us introduce the following notation for the time interpolants on the partition: Given a vector $(u_{0},...,u_{N_\tau })$, we define its backward-constant interpolant $\overline{u}_{\tau }$, its forward-constant interpolant $\underline{u}_{\tau }$, and its piecewise-affine interpolant $\widehat{u}_{\tau }$ on the partition $(t_i)_{i=0}^{N_\tau }$ as

$$\begin{aligned} \overline{u}_\tau (0)&:=u_0, \quad \quad \overline{u}_\tau (t):=u_{i}&\text { if } t\in (t_{i-1},t_i] \quad \text { for } i=1,\dots , N_\tau ,\\ \underline{u}_{\tau }(T)&:=u_{N_\tau }, \quad \; \underline{u}_\tau (t):=u_{i-1}&\text { if } t\in [t_{i-1},t_i) \quad \text { for } i=1,\dots , N_\tau ,\\ \widehat{u}_\tau (0)&:=u_0, \quad \quad \widehat{u}_\tau (t):=\frac{u_i-u_{i-1}}{t_i-t_{i-1}}(t-t_{i-1})+u_{i-1}&\quad \text { if } t\in (t_{i-1},t_i] \quad \text { for } i=1,\dots , N_\tau . \end{aligned}$$

Owing to this notation, we can take the sum in (4.2) for $i=1,\dots ,N_\tau$ and equivalently rewrite the discrete Euler–Lagrange equation in the compact form

4.3 $$\begin{aligned}&\int \limits _0^T\!\!\int \limits _{U } \left( \partial _F W_\varepsilon (\theta {-}\overline{t}_\tau ,\nabla \overline{y}_\tau ){+}\partial _{\dot{F}} R_\varepsilon \left( \theta {-}\overline{t}_\tau ,\nabla \underline{y}_\tau , \nabla \dot{\widehat{y}}_\tau \right) \right) {:}\nabla \overline{z}_\tau \,\textrm{d} x\, \textrm{d} t\nonumber \\&\quad +\int \limits _0^T\!\!\int \limits _U { \mathrm D}H\left( \nabla ^2 \overline{y}_\tau \right) {\vdots } \nabla ^2 \overline{z}_\tau \,\textrm{d} x\, \textrm{d} t= \int \limits _0^T\!\!\int \limits _U f(\theta {-}\overline{t}_\tau ){\cdot } \overline{z}_\tau \,\textrm{d} x\, \textrm{d} t \end{aligned}$$

where $\overline{z}_\tau$ is the backward-constant interpolant of $(z^i)_{i=1}^{N_\tau }$.

From the minimality of $y^i_\tau$ we get that

4.4 $$\begin{aligned}&\int \limits _U W_\varepsilon (\theta {-}t_i,\nabla y^i_\tau )\, \textrm{d} x+\int \limits _UH(\nabla ^2 y^i_\tau )\, \textrm{d} x-\int \limits _U f(\theta {-}t_i){\cdot }y^{i}_\tau \,\textrm{d} x\nonumber \\&\qquad +\tau \int \limits _U R_\varepsilon \left( \theta {-}t_i,\nabla y^{i-1}_\tau ,\frac{\nabla y^i_\tau {-}\nabla y^{i-1}_\tau }{\tau }\right) \,\textrm{d} x\nonumber \\&\quad \le \int \limits _U W_\varepsilon (\theta {-} t_{i-1},\nabla y^{i-1}_\tau )\, \textrm{d} x+\int \limits _UH(\nabla ^2 y^{i-1}_\tau )\,\textrm{d} x-\int \limits _U f(\theta {-} t_{i-1}){\cdot }y^{i-1}_\tau \,\textrm{d} x\nonumber \\&\qquad -\int \limits _U \big (f(\theta {-} t_{i})- f(\theta {-} t_{i-1})\big ){\cdot }y^{i-1}_\tau \,\textrm{d} x\nonumber \\&\qquad - \int \limits _U \big (W_\varepsilon (\theta {-} t_{i-1},\nabla y^{i-1}_\tau ) -W_\varepsilon (\theta {-} t_{i},\nabla y^{i-1}_\tau )\big ) \, \textrm{d} x. \end{aligned}$$

Summing over $i=1,\dots ,n\le N_\tau$ inequality (4.4) we get

4.5 $$\begin{aligned}&\int \limits _U W_\varepsilon (\theta {-}t_n,\nabla y^n_\tau )\, \textrm{d} x+\int \limits _U H(\nabla ^2 y^n_\tau )\,\textrm{d} x-\int \limits _U f(\theta {-}t_n){\cdot }y^n_\tau \,\textrm{d} x\nonumber \\&\qquad +\sum _{i=1}^{n}\tau \int \limits _U R_\varepsilon \left( \theta {-}t_i,\nabla y^{i-1}_\tau ,\frac{\nabla y^i_\tau {-}\nabla y^{i-1}_\tau }{\tau }\right) \,\textrm{d} x\nonumber \\&\quad \le \int \limits _U W_\varepsilon (\theta ,\nabla y_0 )+H(\nabla ^2 y_0)\,\textrm{d} x-\!\int \limits _U \! f(\theta ){\cdot } y_0\,\textrm{d} x\nonumber \\&\qquad -\sum _{i=1}^n\int \limits _U (f(\theta {-}t_i){-}f(\theta {-}t_{i-1})){\cdot }y^{i-1}_\tau \,\textrm{d} x\nonumber \\&\qquad -\sum _{i=1}^n\int \limits _U \big (W_\varepsilon (\theta {-} t_{i-1},\nabla y^{i-1}_\tau ) -W_\varepsilon (\theta {-} t_{i},\nabla y^{i-1}_\tau )\big ) \, \textrm{d} x. \end{aligned}$$

By the growth conditions (2.4)–(2.5) (2.9), (2.13), and (2.14), we hence have that

4.6 $$\begin{aligned}&c_W\Vert \nabla y^n_\tau \Vert _{L^{p}(U; \mathbb {R}^{d\times d})}^p + c_W\left\| \frac{1}{\det \nabla y^n_\tau }\right\| ^q_{L^q(U )}+ c_H \Vert \nabla ^2y^n_\tau \Vert ^p_{L^p(U ; \mathbb {R}^{d\times d\times d} )} \nonumber \\&\qquad + c_{\mathbb {D}} \sum _{i=1}^n\tau \int \limits _U \left| \frac{(\nabla y^i_\tau - \nabla y^{i-1}_\tau )^\top }{\tau } \nabla y^{i-1}_\tau + (\nabla y^{i-1}_\tau )^\top \frac{\nabla y^i_\tau - \nabla y^{i-1}_\tau }{\tau } \right| ^2\, \textrm{d} x- \frac{|U|}{c_W} \nonumber \\&\quad \le \int \limits _U W_\varepsilon (\theta {-}t_n,\nabla y^n_\tau )\, \textrm{d} x+\int \limits _U H(\nabla ^2 y^n_\tau )\,\textrm{d} x+\sum _{i=1}^{n}\tau \int \limits _U R_\varepsilon \left( \theta {-}t_i,\nabla y^{i-1}_\tau ,\frac{\nabla y^i_\tau {-}\nabla y^{i-1}_\tau }{\tau }\right) \,\textrm{d} x\nonumber \\&\quad {\mathop {\le }\limits ^{(4.5)}} \int \limits _U W_\varepsilon (\theta ,\nabla y_0 )+H(\nabla ^2 y_0)\,\textrm{d} x-\!\int \limits _U \! f(\theta ){\cdot } y_0\,\textrm{d} x+\int \limits _U f(\theta {-}t_n){\cdot }y^n_\tau \,\textrm{d} x\nonumber \\&\qquad + \sum _{i=1}^n \int \limits _{t_{i-1}}^{t_i}\int \limits _U \dot{f}(\theta {-}s){\cdot }y^{i-1}_\tau \,\textrm{d} x\, \textrm{d}s  -\sum _{i=1}^n\int \limits _U \big (W_\varepsilon (\theta {-} t_{i-1},\nabla y^{i-1}_\tau ) -W_\varepsilon (\theta {-} t_{i},\nabla y^{i-1}_\tau )\big ) \, \textrm{d} x. \end{aligned}$$

In order to control the right-hand side above, we remark that

$$\begin{aligned}& -\sum _{i=1}^n\int \limits _U \big (W_\varepsilon (\theta {-} t_{i-1},\nabla y^{i-1}_\tau ) -W_\varepsilon (\theta {-} t_{i},\nabla y^{i-1}_\tau )\big ) \, \textrm{d} x\\&\quad {\mathop {=}\limits ^{(2.2)}} \sum _{i=1}^n\int \limits _U \big (h_{ \varepsilon }(\theta {-} t_{i-1})-h_{ \varepsilon }(\theta {-} t_{i})\big )\big (V^a(\nabla y^{i-1}_\tau ) - V^r(\nabla y^{i-1}_\tau ) \big )\, \textrm{d}x\\&\quad {\mathop {\le }\limits ^{(2.4)}} \frac{1}{c_W} \sum _{i=1}^n\int \limits _U \big (h_{ \varepsilon }(\theta {-} t_{i-1})-h_{ \varepsilon }(\theta {-} t_{i})\big )\big (1+|\nabla y^{i-1}_\tau |^p \big )\, \textrm{d}x \end{aligned}$$

where we have also used that $h_{ \varepsilon }(\theta {-} t_{i-1})-h_{ \varepsilon }(\theta {-} t_{i} )\ge 0$. As $h_{ \varepsilon }(\theta {-} t_{i-1})-h_{ \varepsilon }(\theta {-} t_{i} )= 0$ on the complement of

$$\begin{aligned} E_i:=\{x\in U \,: \, \theta (x) \in [t_{i-1}-\varepsilon /2,t_i+\varepsilon /2]\}, \end{aligned}$$

by using $\Vert h'_\varepsilon \Vert _{L^\infty (\mathbb {R})}\le 2/\varepsilon$ (recall (2.1)) and the embedding $L^\infty (U,\mathbb {R}^{d\times d}) \subset W^{1,p}(U,\mathbb {R}^{d\times d})$ we get

$$\begin{aligned}& -\sum _{i=1}^n\int \limits _U \big (W_\varepsilon (\theta {-} t_{i-1},\nabla y^{i-1}_\tau ) -W_\varepsilon (\theta {-} t_{i},\nabla y^{i-1}_\tau )\big ) \, \textrm{d} x\\&\quad \le \frac{c\tau }{\varepsilon }\sum _{i=1}^n|E_i| \left( 1+\Vert \nabla y^{i-1}_\tau \Vert ^p_{L^\infty (U;\mathbb {R}^{d\times d})}\right) \\&\quad \le \frac{c\tau }{\varepsilon }\sum _{i=1}^n|E_i| \left( 1+\Vert \nabla y^{i-1}_\tau \Vert ^p_{L^p(U;\mathbb {R}^{d\times d})} + \Vert \nabla ^2 y^{i-1}_\tau \Vert ^p_{L^p(U;\mathbb {R}^{d\times d \times d})}\right) . \end{aligned}$$

Together with (2.14)–(2.15), this allows to deduce from inequality (4.6) that

4.7 $$\begin{aligned}&c_W\Vert \nabla y^n_\tau \Vert ^{ p}_{L^{ p}(U; \mathbb {R}^{d\times d} )}+ c_W\left\| \frac{1}{\det \nabla y^n_\tau }\right\| ^q_{L^q(U )}+ c_H \Vert \nabla ^2y^n_\tau \Vert ^p_{L^p(U ; \mathbb {R}^{d\times d\times d} )} \nonumber \\&\qquad + c_{\mathbb {D}} \sum _{i=1}^n\tau \int \limits _U \left| \frac{(\nabla y^i_\tau - \nabla y^{i-1}_\tau )^\top }{\tau } \nabla y^{i-1}_\tau (\nabla y^{i-1}_\tau )^\top \frac{\nabla y^i_\tau - \nabla y^{i-1}_\tau }{\tau } \right| ^2\, \textrm{d} x\nonumber \\&\quad \le c + c\Vert y^n_\tau \Vert _{L^2(U;\mathbb {R}^d)} + c\sum _{i=1}^n\tau \Vert y^{i-1}_\tau \Vert _{L^2(U;\mathbb {R}^d)} \nonumber \\&\qquad + \frac{c\tau }{\varepsilon }\sum _{i=1}^{n} |E_i|\left( 1+ \Vert \nabla y^{i-1}_\tau \Vert ^p_{L^p(U;\mathbb {R}^{d\times d})}+ \Vert \nabla ^2 y^{i-1}_\tau \Vert ^p_{L^p(U;\mathbb {R}^{d\times d\times d})}\right) . \end{aligned}$$

For $\tau <\varepsilon$ one has that $\cup _{i=1}^{N_\tau } E_i$ covers $\Omega (T)$ multiple times. In particular, we have that

4.8 $$\begin{aligned} \sum _{i=1}^{N_\tau }|E_i| \le \left( \frac{\varepsilon +\tau }{\tau } +1\right) |\Omega (T)|. \end{aligned}$$

Hence, by the Poincaré inequality and the Discrete Gronwall Lemma [27, (C.2.6), p. 534] we find the bound

4.9 $$\begin{aligned}&\max _n \Vert y^n_\tau \Vert _{W^{2,p}(U,\mathbb {R}^d)}^{ p} +\sum _{i=1}^{N_\tau }\tau \left\| \frac{(\nabla y^i_\tau {-} \nabla y^{i-1}_\tau )^\top }{\tau } \nabla y^{i-1}_\tau + (\nabla y^{i-1}_\tau )^\top \frac{\nabla y^i_\tau {-} \nabla y^{i-1}_\tau }{\tau } \right\| ^2_{L^2(U;\mathbb {R}^{d\times d})} \nonumber \\&\quad \le c \, \textrm{exp} \left( \frac{c\tau }{\varepsilon } \sum _{i=i}^{N_\tau }|E_i| \right) {\mathop {\le }\limits ^{(4.8)}} c \, \textrm{exp} \left( \frac{c\tau }{\varepsilon } \left( \frac{\varepsilon + \tau }{\tau }+1\right) |\Omega (T)|\right) \le c \, \textrm{exp}(c\tau /\varepsilon ), \end{aligned}$$

where we also used the fact that $\Omega (T) \subset \subset U$.

By the Sobolev embedding of $W^{2,p}(U;\mathbb {R}^d)$ into $C^{1-d/p}(U;\mathbb {R}^d)$ and the classical result of [22, Thm. 3.1] we get

4.10 $$\begin{aligned} \det \nabla \overline{y}_\tau \ge c_\varepsilon >0 \ \ \text {in} \ \ [0,T]\times \overline{U} \end{aligned}$$

where the constant $c_\varepsilon$ depends on the bound in (4.9).

By the Poincaré inequality and the generalization of Korn’s first inequality by [40] and [43, Thm. 2.2], also using (4.10) we have that

$$\begin{aligned} \Vert \nabla \dot{\widehat{y}}_\tau \Vert ^{ 2}_{ L^2(0,T; L^2( Q;\mathbb {R}^{d\times d} ))}\le c_\varepsilon ' \int \limits _0^T\ \Vert \nabla \dot{\widehat{y}}_\tau ^\top \nabla \underline{y}_\tau + \nabla \underline{y}_\tau ^\top \nabla \dot{\widehat{y}}_\tau \Vert ^2_{L^2(U;\mathbb {R}^{d\times d})} \, \textrm{d}s {\mathop {\le }\limits ^{(4.9)}}c_\varepsilon ' c \, \textrm{exp}(c\tau /\varepsilon ) \end{aligned}$$

where the constant $c_\varepsilon '$ depends on the bound (4.9) and on the constant $c_\varepsilon$ in (4.10). Again by the Poincaré inequality, this time applied to $\dot{y}$, we get that

4.11 $$\begin{aligned} \Vert {\widehat{y}}_\tau \Vert _{H^1(0,T;H^1(U; \mathbb {R}^d ))}\le c_\varepsilon ' c \, \textrm{exp}(c\tau /\varepsilon ). \end{aligned}$$

By using these estimates, as $\tau \rightarrow 0$, up to not relabeled subsequences we get

4.12 $$\begin{aligned}&\overline{y}_\tau ,\,\underline{y}_\tau {\mathop {\rightharpoonup }\limits ^{*}} y \quad \text {weakly-}*\, \text {in} \ \ L^{\infty }(0,T;W^{2,p}(U ;\mathbb {R}^d)), \end{aligned}$$

4.13 $$\begin{aligned}&\nabla \dot{\widehat{y}}_\tau \rightharpoonup \nabla \dot{y} \quad \text {weakly in} \ \ L^{2}( Q;\mathbb {R}^d), \end{aligned}$$

4.14 $$\begin{aligned}&\nabla \widehat{y}_\tau \rightarrow \nabla y \quad \text {strongly in} \ \ C^{0,\alpha }( \overline{Q} ;\mathbb {R}^d) \end{aligned}$$

for some $\alpha \in (0,1)$. In particular, from the convergences above we also get $\det \nabla \overline{y}_\tau \rightarrow \det \nabla y$ uniformly. In combination with the lower bound (4.10), this implies that $\nabla y \in \textrm{GL}_+(d)$ everywhere, hence *y* is admissible, namely, $y(t,\cdot ) \in \mathcal {A}$ for every $t\in (0,T)$.

We now pass to the limit in the time-discrete Euler–Lagrange equation (4.3). Let $z\in C^\infty (\overline{Q};\mathbb {R}^d)$ with $z=0$ on $\Sigma _D$ be given and let $(z^i_\tau )_{i=1}^{N_\tau }\in \mathcal {A}$ be such that $\overline{z}_\tau \rightarrow z$ strongly in $L^\infty (0,T;W^{2,p}(U;\mathbb {R}^d))$. By (2.14) we have

4.15 $$\begin{aligned} \int \limits _0^T\!\!\int \limits _U f(\theta {-}\overline{t}_\tau ){\cdot } \overline{z}_\tau \,\textrm{d} x\,\textrm{d} t\rightarrow \int \limits _0^T\!\!\int \limits _U f(\theta {-}t){\cdot }z\,\textrm{d} x\,\textrm{d} t. \end{aligned}$$

As $h_\varepsilon (\theta (x){-}\overline{t}_\tau (t))\rightarrow h_\varepsilon (\theta (x){-}t)$ for almost every $(t,x)\in Q$, the dissipation term goes to the limit as follows

4.16 $$\begin{aligned}&\int \limits _0^T\!\!\int \limits _U \partial _{\dot{F}} R_\varepsilon \left( \theta {-}\overline{t}_\tau ,\nabla \underline{y}_\tau ,\nabla \dot{\widehat{y} }_\tau \right) {:}\nabla \overline{z}_\tau \,\textrm{d} x\,\textrm{d} t\nonumber \\&\quad =2\int \limits _0^T\!\!\int \limits _U (1{-}h_\varepsilon (\theta {-}\overline{t}_\tau ))\nabla \underline{y}_\tau \left( \mathbb {D}^{ a}(\nabla \underline{y}_\tau ^\top \nabla \underline{y}_\tau )(\nabla \dot{\widehat{y} }_\tau ^\top \nabla \underline{y}_\tau {+}\nabla \underline{y}_\tau ^\top \nabla \dot{\widehat{y} }_\tau )\right) {:}\nabla \overline{z}_\tau \,\textrm{d} x\,\textrm{d} t\nonumber \\&\qquad +2\int \limits _0^T\!\!\int \limits _U h_\varepsilon (\theta {-}\overline{t}_\tau )\nabla \underline{y}_\tau \left( \mathbb {D}^r(\nabla \underline{y}_\tau ^\top \nabla \underline{y}_\tau )(\nabla \dot{\widehat{y} }_\tau ^\top \nabla \underline{y}_\tau {+}\nabla \underline{y}_\tau ^\top \nabla \dot{\widehat{y} }_\tau )\right) {:}\nabla \overline{z}_\tau \,\textrm{d} x\,\textrm{d} t\nonumber \\&\quad \rightarrow 2\int \limits _0^T\!\!\int \limits _U (1{-}h_\varepsilon (\theta {-}t))\nabla y \left( \mathbb {D}^{ a}(\nabla y^\top \nabla y)(\nabla \dot{y}^\top \nabla y{+}\nabla y^\top \nabla \dot{y})\right) {:}\nabla z\,\textrm{d} x\,\textrm{d} t\nonumber \\&\qquad + 2\int \limits _0^T\!\!\int \limits _U h_\varepsilon (\theta {-}t)\nabla y \left( \mathbb {D}^r(\nabla y^\top \nabla y)(\nabla \dot{y}^\top \nabla y{+}\nabla y^\top \nabla \dot{y})\right) {:}\nabla z\,\textrm{d} x\,\textrm{d} t\end{aligned}$$

4.17 $$\begin{aligned}&=\int \limits _0^T\!\!\int \limits _U \partial _{\dot{F}} R_\varepsilon \left( \theta {-}t,\nabla y,\nabla \dot{y}\right) {:}\nabla z \,\textrm{d} x\,\textrm{d} t \end{aligned}$$

where we used (2.12) and convergences (4.12)–(4.14). Moreover, we also have

4.18 $$\begin{aligned} \int \limits _0^T\int \limits _{U }\partial _F W_\varepsilon (\theta {-}\overline{t}_\tau ,\nabla \overline{y}_\tau ) {:} \nabla \overline{z}_\tau \,\textrm{d} x\,\textrm{d} t\rightarrow \int \limits _0^T\int \limits _{U }\partial _F W_\varepsilon (\theta {-}t,\nabla y) {:} \nabla {z} \,\textrm{d} x\,\textrm{d} t \end{aligned}$$

by (2.3) and convergences (4.12) and (4.14).

For the convergence of the second-gradient term we reproduce in this setting the argument from [25]. Given the limit *y*, let $(w_{\tau }^i)_{i=1}^{N_\tau }\in \mathcal {A}$ be such that $\overline{w}_{\tau } \rightarrow y$ strongly in $L^\infty (0,T;W^{2,p}(U;\mathbb {R}^d))$. We consider the test functions $\overline{z}_{\tau } {:}{=}\overline{w}_{\tau }-\overline{y}_{\tau }$ in the time-discrete Euler–Lagrange equation (4.3). Convergences (4.12)–(4.13) entail that $\overline{z}_{\tau }\rightarrow 0$ strongly in $L^\infty (0,T;H^{1}(U;\mathbb {R}^d))$ and $\overline{z}_{\tau }\rightharpoonup 0$ weakly-$*$ in $L^{ \infty } (0,T;W^{2,p} (U;\mathbb {R}^d))$. Let us now compute

4.19 $$\begin{aligned}&\int \limits _0^T\!\!\int \limits _U({ \mathrm D}H(\nabla ^2 y)-{ \mathrm D} H(\nabla ^2 \overline{y}_\tau )){\vdots }(\nabla ^2 y -\nabla ^2 \overline{y}_\tau )\,\textrm{d} x\,\textrm{d} t\nonumber \\&\quad = \int \limits _0^T\!\!\int \limits _U({ \mathrm D}H(\nabla ^2 y)-{ \mathrm D} H(\nabla ^2 \overline{y}_\tau )){\vdots }(\nabla ^2 y - \nabla ^2 \overline{w}_{\tau })\,\textrm{d} x\,\textrm{d} t\nonumber \\&\qquad +\int \limits _0^T\!\!\int \limits _U({ \mathrm D}H(\nabla ^2 y)-{ \mathrm D} H(\nabla ^2 \overline{y}_\tau )){\vdots }\nabla ^2 \overline{z}_{\tau }\,\textrm{d} x\,\textrm{d} t. \end{aligned}$$

As $\nabla ^2 \overline{w}_{\tau } \rightarrow \nabla ^2 y$ strongly in $L^{p}( Q;\mathbb {R}^{d\times d \times d})$ and $\textrm{D} H (\nabla ^2 \overline{y}_\tau )$ is bounded in $L^{p'}( Q;\mathbb {R}^{d\times d \times d})$ by (2.9), the first integral in the right-hand side above converges to 0 as $\tau \rightarrow 0$. Hence, passing to the $\limsup$ in (4.19), by the Euler–Lagrange equation (4.3) and convergences (4.14)–(4.18) we find that

4.20 $$\begin{aligned}&\limsup _{\tau \rightarrow 0} \int \limits _0^T\!\!\int \limits _U({ \mathrm D}H(\nabla ^2 y)-{ \mathrm D} H(\nabla ^2 \overline{y}_\tau )){\vdots }(\nabla ^2y - \nabla ^2 \overline{y}_\tau ) \,\textrm{d} x\,\textrm{d} t\nonumber \\&\quad = \limsup _{\tau \rightarrow 0} \int \limits _0^T\!\!\int \limits _U({ \mathrm D}H(\nabla ^2 y)-{ \mathrm D} H(\nabla ^2 \overline{y}_\tau )){\vdots }\nabla ^2 \overline{z}_{\tau }\,\textrm{d} x\,\textrm{d} t\nonumber \\&\quad =\limsup _{\tau \rightarrow 0}\Bigg (\int \limits _0^T\!\!\int \limits _U{ \mathrm D} H(\nabla ^2 y){\vdots }\nabla ^2 \overline{z}_{\tau }\, \textrm{d} x\, \textrm{d} t- \int \limits _0^T\!\!\int \limits _U f(\theta {-}\overline{t}_\tau ){\cdot } \overline{z}_{\tau }\,\textrm{d} x\,\textrm{d} t\nonumber \\&\qquad +\int \limits _0^T\!\!\int \limits _U\left( \partial _F W_\varepsilon (\theta {-}\overline{t}_\tau , \nabla \overline{y}_\tau )+\partial _{\dot{F}}R_\varepsilon \left( \theta {-}\overline{t}_\tau , \nabla \underline{y}_\tau , \nabla \dot{\widehat{y}}_\tau \right) \right) {:}\nabla \overline{z}_{\tau }\,\textrm{d} x\,\textrm{d} t \Bigg )=0 \end{aligned}$$

The coercivity (2.9) then implies that

$$\begin{aligned} \nabla ^2 \overline{y}_\tau \rightarrow \nabla ^2 y \quad \text {strongly in} \ \ L^p(Q; \mathbb {R}^{d\times d\times d}) \end{aligned}$$

and thus

$$\begin{aligned} \textrm{D} H(\nabla ^2 \overline{y}_\tau )\rightarrow { \mathrm D} H(\nabla ^2 {y}) \quad \text { strongly in } L^{p'}(Q; \mathbb {R}^{d\times d \times d}). \end{aligned}$$

Passing to the limit as $\tau \rightarrow 0$ in (4.3) we then find (2.18).

In order to prove the bound (4.1), we simply pass to the limit as $\tau \rightarrow 0$ in (4.9) and obtain

$$\begin{aligned} \Vert y \Vert _{L^\infty (0,T;W^{2,p}(U;\mathbb {R}^d))}^{ p} + \Vert \nabla \dot{y}^\top \nabla y+ \nabla y^\top \nabla \dot{y} \Vert _{L^2(Q;\mathbb {R}^{d\times d})}^{ 2} \le c \end{aligned}$$

independently of $\varepsilon$. Following again [22, Thm. 3.1] we have that $\det \nabla y \ge c>0$ independently of $\varepsilon$. By [40] and [43, Thm. 2.2] this ensures that

$$\begin{aligned} \Vert \nabla \dot{y} \Vert _{L^2(Q;\mathbb {R}^{d\times d})}^2\le c \Vert \nabla \dot{y}^\top \nabla y+ \nabla y^\top \nabla \dot{y} \Vert _{L^2(Q;\mathbb {R}^{d\times d})}^2\le c \end{aligned}$$

independently of $\varepsilon$. Hence, (4.1) follows by the Poincaré inequality.$\square$

Before moving to the proof of Theorem 2.1 in the diffused-interface case $\varepsilon >0$, let us recall a well-posedness result for the growth subproblem, see [33, Thm. 3.15].

### Proposition 4.2

(Well-posedness of the growth problem) Assume to be given $\widehat{\gamma }\in C(\mathbb {R}^d)$ with $c_\gamma \le \widehat{\gamma }(\cdot ) \le C_\gamma$ for some $0<c_\gamma \le C_\gamma$ and $\Omega _0\subset \mathbb {R}^d$ nonempty, open, and bounded. Then, there exists a unique viscosity solution to

4.21 $$\begin{aligned}&\widehat{\gamma }(x) |\nabla (-\theta )(x)|=1 \quad \text {in} \ \ \mathbb {R}^d \setminus \overline{\Omega _0}, \end{aligned}$$

4.22 $$\begin{aligned}&\theta =0 \quad \text {in} \ \ {\Omega _0}. \end{aligned}$$

Moreover, $$\theta \in C^{0,1}(\mathbb {R}^d)$$ with

4.23 $$\begin{aligned} 0<\frac{1}{C_\gamma } \le |\nabla \theta (x)| \le \frac{1}{c_\gamma } \ \ \text {for a.e.} \ \ x\in \mathbb {R}^d, \end{aligned}$$

and we have that

4.24 $$\begin{aligned} \frac{\operatorname {dist}(x,{\Omega _0})}{C_\gamma }\le \theta (x) \le \frac{\operatorname {dist}(x,{\Omega _0})}{c_\gamma } \quad \forall x \in \mathbb {R}^d \setminus \overline{\Omega _0}. \end{aligned}$$

We are now ready to prove Theorem 2.1 in the diffused-interface case $\varepsilon >0$. As announced, the proof hinges on an iterative construction. To start with, let us remark that $y_0$ from (2.15) is such that $\nabla y_0$ is Hölder continuous. In particular, the mapping ${\tilde{\gamma }}: \overline{U} \rightarrow (0,\infty )$ defined by

$$\begin{aligned} {\tilde{\gamma }} ( x):= \gamma (y_0( x),\nabla y_0 ( x))\quad \forall x\in \overline{U} \end{aligned}$$

is Hölder continuous, as well. Letting $\widehat{\gamma }$ be any continuous extension of ${\tilde{\gamma }}$ to $\mathbb {R}^d$ with $c_\gamma \le \widehat{\gamma }(\cdot )\le C_\gamma$, we can use Proposition 4.2 and find $\theta _0 \in C({\overline{U}} )$ solving

$$\begin{aligned}&\gamma (y_0( x),\nabla y_0( x))|\nabla (-\theta _0)(x)|=1\quad \text {in} \ U \setminus \overline{\Omega _0},\\&\quad \theta _0=0 \quad \text {in} \ \Omega _0 \end{aligned}$$

in the viscosity sense, with (4.23) and (4.24) holding in ${\overline{U}}$. Note that (4.24) in particular implies that

$$\begin{aligned} \Omega ^0(T)=\{x\in U \ | \ \theta _0(x)<T\} \subset \Omega _0 +B_{C_\gamma T}{\mathop {\subset \subset }\limits ^{(2.17)}} U. \end{aligned}$$

By applying Proposition 4.1 for $\theta =\theta _0$ we find $y^1 \in L^{\infty }(0,T;W^{2,p}(U;\mathbb {R}^d))\cap H^1(0,T;H^1(U;\mathbb {R}^d))$.

This can be iterated as follows: For all $k\ge 1$, given $y^k \in L^{\infty }(0,T;W^{2,p}(U;\mathbb {R}^d))\cap H^1(0,T;H^1(U;\mathbb {R}^d))$ we define $\theta ^k\in C({\overline{U}} )$ to be a viscosity solution to

$$\begin{aligned}&\gamma (y^k(\theta ^k (x) {\wedge } T ,x),\nabla y^k(\theta ^k(x) {\wedge } T ,x))|\nabla (-\theta ^k) (x)|=1\quad \text {in} \ U \setminus \overline{\Omega _0},\\&\quad \theta ^k =0 \quad \text {in} \ \Omega _0 \end{aligned}$$

with (4.23) and (4.24) holding in ${\overline{U}}$. The existence of such a viscosity solution follows again from Proposition 4.2 as the mapping on $\overline{U}$ defined as

$$\begin{aligned} x \mapsto \gamma (y^k(\theta ^k (x) {\wedge } T,x),\nabla y^k(\theta ^k(x) {\wedge } T,x))\quad \forall x \in \overline{U} \end{aligned}$$

may be extended to a continuous mapping $\widehat{\gamma }$ on $\mathbb {R}^d$ with $c_\gamma \le \widehat{\gamma }(\cdot )\le C_\gamma$. Note again that (4.24) implies that

4.25 $$\begin{aligned} \Omega ^k( T ) :=\{x\in U \ | \ \theta ^k(x)< T \} \subset \Omega _0 +B_{C_\gamma T}{\mathop {\subset \subset }\limits ^{(2.17)}} U. \end{aligned}$$

Inclusion (4.25) in particular guarantees that the accreting phase defined by $\theta ^k$ remains at positive distance from the boundary $\partial U$, independently of $\varepsilon$ and *k*.

Given such $\theta ^k$, we define $y^{k+1} \in L^{\infty }(0,T;W^{2,p}(U;\mathbb {R}^d))\cap H^1(0,T;H^1(U;\mathbb {R}^d))$ by Proposition 4.1 applied for $\theta =\theta ^k$.

Bounds (4.1) and (4.23) ensure that the sequence $(y^k, \theta ^k)_{k\in \mathbb {N}}$ defined by this iterative procedure is (possibly not unique but nonetheless) uniformly bounded in

$$\begin{aligned} \left( L^{\infty }(0,T;W^{2,p}(U;\mathbb {R}^d))\cap H^1(0,T;H^1(U;\mathbb {R}^d))\right) \times C^{0,1}({\overline{U}}). \end{aligned}$$

As $(\theta ^k)_{i\in \mathbb {N}}$ are uniformly Lipschitz continuous, by the Ascoli–Arzelà and the Banach–Alaoglu Theorems, possibly passing to not relabeled subsequences, one can find a pair $(y, \theta )$ such that

4.26 $$\begin{aligned}&y^k {\mathop {\rightharpoonup }\limits ^{*}} y \quad \text {weakly-* in} \ L^{\infty }(0,T;W^{2,p}(U ;\mathbb {R}^d))\cap H^1(0,T;H^1(U ;\mathbb {R}^d)), \end{aligned}$$

4.27 $$\begin{aligned}&y^k \rightarrow y \quad \text {strongly in} \ C^{1,\alpha }( \overline{Q} ;\mathbb {R}^d), \end{aligned}$$

4.28 $$\begin{aligned}&\theta ^k \rightarrow \theta \quad \text {strongly in} \ C( \overline{U}) \end{aligned}$$

for some $\alpha \in (0,1)$ and $\theta$ fulfills (4.23) and (4.24) in ${\overline{U}}$. As $(y^k)_{k\in \mathbb {N}}$ are uniformly Hölder continuous and $\gamma$ is Lipschitz continuous, by (2.16) we have

$$\begin{aligned}&|\gamma (y^k(\theta ^k(x) {\wedge } T ,x),\nabla y^k(\theta ^k(x) {\wedge } T ,x ) )- \gamma (y^j(\theta ^j(x) {\wedge } T ,x),\nabla y^j(\theta ^j(x) {\wedge } T ,x ))|\\&\quad \le c|y^k(\theta ^k(x) {\wedge } T ,x)-y^j(\theta ^j(x) {\wedge } T ,x)|+c|\nabla y^k(\theta ^k(x) {\wedge } T ,x)-\nabla y^j(\theta ^j(x) {\wedge } T ,x)|\\&\quad \le c|y^k(\theta ^k(x) {\wedge } T ,x)-y^j(\theta ^k(x) {\wedge } T ,x)|+c|\nabla y^k(\theta ^k(x) {\wedge } T ,x)-\nabla y^j(\theta ^k(x) {\wedge } T ,x)|\\&\qquad + c|y^j(\theta ^k(x) {\wedge } T ,x)-y^j(\theta ^j(x) {\wedge } T ,x)|+c|\nabla y^j(\theta ^k(x) {\wedge } T ,x)-\nabla y^j(\theta ^j(x) {\wedge } T ,x)|\\&\quad \le c\Vert y^k - y^j\Vert _{C^1( {\overline{Q}} ; \mathbb {R}^d ) } + c \Vert \theta ^k - \theta ^j\Vert ^\alpha _{C(\overline{U})} \quad \forall \ x \in {\overline{U}}. \end{aligned}$$

Together with (4.27)–(4.28), this proves that $x \mapsto \gamma (y^k(\theta ^k(x) {\wedge } T,x),\nabla y^k(\theta ^k(x) {\wedge } T,x ) )$ converges to $x \mapsto \gamma (y(\theta (x) {\wedge } T,x),\nabla y(\theta (x) {\wedge } T,x ) )$ uniformly in ${\overline{U}}$. By the stability of the eikonal equation with respect to the uniform convergence of the data, see, e.g., [23, Prop. 1.2], $\theta$ satisfies (2.19)–(2.20) with coefficient $x \mapsto \gamma (y(\theta (x) {\wedge } T,x),\nabla y(\theta (x) {\wedge } T,x ) )$. Moreover, since bound (4.1) is independent of $\theta$, following the argument of the proof of Proposition 4.1, we can pass to the limit in the Euler–Lagrange equation (2.18) and conclude the proof of Theorem 2.1 in the case $\varepsilon >0$.

## Proof of Theorem 2.1: sharp-interface case

The existence of weak/viscosity solutions in the sharp-interface case $\varepsilon =0$ is obtained by passing to the limit as $\varepsilon \rightarrow 0$ in sequences of weak/viscosity solutions $(y_\varepsilon ,\theta _\varepsilon )$ of the diffused-interface problem.

Notice at first that $\theta _\varepsilon$ are uniformly Lipschitz continuous, see (4.23). Bound (4.1) is independent of $\varepsilon$ and implies that there exist not relabeled subsequences such that

5.1 $$\begin{aligned}&y_\varepsilon {\mathop {\rightharpoonup }\limits ^{*}} y \quad \text {weakly-}*\,\text { in} \ L^{\infty }(0,T;W^{2,p}(U ;\mathbb {R}^d))\cap H^1(0,T;H^1(U ;\mathbb {R}^d)), \end{aligned}$$

5.2 $$\begin{aligned}&y_\varepsilon \rightarrow y \quad \text {strongly in} \ C^{1,\alpha }(\overline{Q};\mathbb {R}^d), \end{aligned}$$

5.3 $$\begin{aligned}&\theta _\varepsilon \rightarrow \theta \quad \text {strongly in} \ C(\overline{U}) \end{aligned}$$

for some $$\alpha \in (0,1)$$.

Let us now prove that we can pass to the limit $\varepsilon \rightarrow 0$ in equation (2.18). The convergence of the loading is straightforward. Moreover, the level sets $\{ \theta (x)=t\}$ have Lebesgue measure zero by (4.23). Hence, by the assumptions (2.1) on $h_\varepsilon$ and the uniform convergence (5.3) of $(\theta _\varepsilon )_\varepsilon$, we have that

$$\begin{aligned} h_\varepsilon (\theta _\varepsilon (x){-}t)\rightarrow h_0(\theta (x){-}t) \quad \text { for a.e. } (t,x)\in Q, \end{aligned}$$

and that $(t,x)\mapsto h_\varepsilon (\theta _\varepsilon (x){-}t)$ converges to $(t,x)\mapsto h_0(\theta (x){-}t)$ strongly in $L^2( Q )$. On the other hand, by (2.3) and convergence (5.2), for all $(t,x)\in Q$ and $i= a,\, r,\, J$, we have that

$$\begin{aligned} |\partial _F V^i (\nabla y_\varepsilon )| \le c \quad \text { and } \quad \partial _F V^i (\nabla y_\varepsilon )\rightarrow \partial _F V^i (\nabla y). \end{aligned}$$

Fix $z\in C^{\infty }([0,T]\times \overline{U };\mathbb {R}^d)$ with $z=0$ on $\Sigma _D$. By Lebesgue’s Dominated Convergence Theorem we get

$$\begin{aligned}&\int \limits _0^T\!\!\int \limits _U \partial _F W_\varepsilon (\theta _\varepsilon {-}t,\nabla y_\varepsilon ){:}\nabla z \,\textrm{d} x\,\textrm{d} t\\&\quad =\int \limits _0^T\!\!\int \limits _U \Big (h_\varepsilon (\theta {-}t)\partial _F V^r (\nabla y_\varepsilon ) + (1-h_\varepsilon (\theta {-}t))\partial _F V^a (\nabla y_\varepsilon ) + \partial _F V^{J}(\nabla y_\varepsilon )\Big ) {:}\nabla z\,\textrm{d} x\,\textrm{d} t\\&\quad \rightarrow \int \limits _0^T\!\!\int \limits _U \Big (h_0(\theta {-}t)\partial _F V^r (\nabla y) + (1{-}h_0(\theta {-}t))\partial _F V^a (\nabla y) + \partial _F V^{J}(\nabla y)\Big ) {:}\nabla z \,\textrm{d} x\,\textrm{d} t\\&\quad =\int \limits _0^T\!\!\int \limits _U \partial _F W_0(\theta {-}t,\nabla y) {:}\nabla z \,\textrm{d} x\,\textrm{d} t\end{aligned}$$

Furthermore, by using convergence (5.1), we get

$$\begin{aligned}&\int \limits _0^T\!\!\int \limits _U \partial _{\dot{F}} R_\varepsilon (\theta _\varepsilon {-}t,\nabla y_\varepsilon , \nabla \dot{y}_\varepsilon ){:}\nabla z \,\textrm{d} x\,\textrm{d} t\\&\quad \rightarrow \int \limits _0^T\!\!\int \limits _U\Big ( h_0(\theta {-}t)\partial _{\dot{F}}R^r(\nabla y, \nabla \dot{y})+ (1{-}h_0(\theta {-}t))\partial _{\dot{F}}R^{a}(\nabla y, \nabla \dot{y})\Big ) {:}\nabla z \,\textrm{d} x\,\textrm{d} t\\&\quad = \int \limits _0^T\!\!\int \limits _U \partial _{\dot{F}} R_0(\theta {-}t,\nabla y, \nabla \dot{y}){:}\nabla z \,\textrm{d} x\,\textrm{d} t. \end{aligned}$$

In order to prove the convergence of the second-order term, we set $z_\varepsilon =y-y_\varepsilon$ and recall that $z_\varepsilon \rightarrow 0$ strongly in $L^\infty (0,T;H^1(U;\mathbb {R}^d))$ and $z_\varepsilon {\mathop {\rightharpoonup }\limits ^{*}} 0$ weakly-$*$ in $L^\infty (0,T;W^{2,p}(U;\mathbb {R}^d))$ in order to obtain

$$\begin{aligned}&\limsup _{\tau \rightarrow 0} \int \limits _0^T\!\!\int \limits _U({ \mathrm D}H(\nabla ^2 y)-{ \mathrm D} H(\nabla ^2 y_\varepsilon )){\vdots }\nabla ^2 z_\varepsilon \,\textrm{d} x\,\textrm{d} t\\&\quad =\limsup _{\varepsilon \rightarrow 0}\Bigg (\int \limits _0^T\!\!\int \limits _U{ \mathrm D} H(\nabla ^2 y ){\vdots }\nabla ^2 z_\varepsilon -f(\theta {-}t){\cdot }z_\varepsilon \,\textrm{d} x\,\textrm{d} t\\&\qquad +\int \limits _0^T\!\!\int \limits _U\Big (\partial _F W_\varepsilon (\theta {-}t, \nabla y_\varepsilon ) {:}\nabla z_\varepsilon +\partial _{\dot{F}}R_\varepsilon \left( \theta {-}t, \nabla y_\varepsilon , \nabla \dot{y}_\varepsilon \right) {:}\nabla z_\varepsilon \Big )\,\textrm{d} x\,\textrm{d} t\Bigg )=0 \end{aligned}$$

Owing to (2.10) this proves that $\nabla ^2 y_\varepsilon \rightarrow \nabla ^2 y$ strongly in $L^p(Q;\mathbb {R}^{d\times d\times d})$. We hence have that $\textrm{D}H(\nabla ^2 y_\varepsilon ) \rightarrow \textrm{D}H(\nabla ^2 y)$ strongly in $L^{p'}(Q;\mathbb {R}^{d\times d\times d})$, as well, and we can pass to the limit as $\varepsilon \rightarrow 0$ in (2.18).

In order to conclude the proof, we are left to check that $\theta$ is a viscosity solution to (2.19). This however readily follows as $x\mapsto \gamma (y_\varepsilon (\theta _\varepsilon (x)\wedge T,x),\nabla y_\varepsilon (\theta _\varepsilon (x)\wedge T,x))$ converges to $x\mapsto \gamma (y(\theta (x)\wedge T,x),\nabla y(\theta (x)\wedge T,x))$ uniformly and the eikonal problem is stable under uniform convergence of the data [23, Prop. 1.2].

Before closing this section, let us explicitly remark that indeed Proposition 2.1 actually holds in the case $\varepsilon =0$, as well. In order to check it, one would need a slightly different, and indeed simpler, a-priori estimate on the time-discrete solutions. Based on such result, one could argue as in Sect. 4 by the same iterative procedure in order to obtain an alternative proof of Theorem 2.1 in the sharp-interface case.
